# Impact of *Phytophthora agathidicida* infection on canopy and forest floor plant nutrient concentrations and fluxes in a kauri‐dominated forest

**DOI:** 10.1002/ece3.7326

**Published:** 2021-03-19

**Authors:** Luitgard Schwendenmann, Beate Michalzik

**Affiliations:** ^1^ School of Environment University of Auckland Auckland New Zealand; ^2^ Soil Science Institute of Geography Friedrich Schiller University Jena Jena Germany; ^3^ German Centre for Integrative Biodiversity Research (iDiv) Halle‐Jena‐Leipzig Leipzig Germany

**Keywords:** forest floor leachate, macro‐, micro‐, and beneficial nutrients, nutrient cycling, *Phytophthora*, plant pathogens, stemflow, throughfall

## Abstract

Kauri dieback, caused by *Phytophthora agathidicida*, is a biotic disturbance that poses a recent threat to the survival of kauri (*Agathis australis*) forests in New Zealand. Previous studies have shown that throughfall and stemflow play an important role in the kauri forests’ internal nutrient cycle. However, the effects of *P. agathidicida* infection on canopy and forest floor nutrient fluxes in kauri forests remain unknown. Here, we measured throughfall, stemflow and forest floor water yield, nutrient (potassium, calcium, magnesium, manganese, silicon, sulfur, sodium, iron) concentrations and fluxes of ten kauri trees differing in soil *P. agathidicida* DNA concentration, and health status. We did not observe an effect of soil *P. agathidicida* DNA concentration on throughfall, stemflow, and forest floor water yield. Throughfall and forest floor nutrient concentrations and fluxes decreased (up to 50%) with increasing soil *P. agathidicida* DNA concentration. We found significant effects on potassium and manganese fluxes in throughfall; calcium and silicon fluxes in forest floor leachate. A decline in canopy and forest floor nutrient fluxes may result in soil nutrient imbalances, which in turn may negatively impact forest productivity and may increase the susceptibility of trees to future pathogen infection in these ecologically unique kauri forests. Given our findings and the increasing spread of *Phytophthora* species worldwide, research on the underlying physiological mechanisms linking dieback and plant–soil nutrient fluxes is critical.

## Graphical abstract

First study on nutrient fluxes in a *Phytophthora agathidicida*‐infected forest. Canopy and forest floor nutrient fluxes decreased with increasing *P. agathidicida* infection. Decline in plant nutrient supply may increase susceptibility to future pathogen infection.

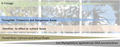

## 1 INTRODUCTION

Natural disturbances can substantially alter a range of ecosystem processes and related functions, and consequently ecosystem services (Rouault et al., 2006; Schowalter et al., 2018; Thom & Seidl, 2016). Insects and pathogens are major natural biotic disturbance agents and often an integral part of forest dynamics (Dale et al., 2001; Kautz et al., 2017; Wardle & Bardgett, 2008). Biotic disturbances can disrupt ecosystems' structure, composition, and function at the stand and landscape scale (Burdon & Laine, 2019; Flower & Gonzalez‐Meler, 2015; Seidl et al., 2017). Kauri dieback, caused by *Phytophthora agathidicida*, is one such recent threat that endangers the survival of the ecologically important conifer species *Agathis australis* (kauri) in New Zealand. *Phytophthora* species (Oomycota, Stramenopila) are soil‐, water‐, or airborne plant pathogens that pose major challenges to global biosecurity, and some *Phytophthora* species are threats to forest ecosystems across the globe (Jung et al., 2018). A changing climate may increase the pressure on forests, as changes in temperature and rainfall patterns may result in changes to the biogeographic range of plant pathogens (Shaw & Osborne, 2011) and may increase outbreaks (Jamieson et al., 2012). *Phytophthora* species are responsible for the mortality of several tree species in Europe, North America, and Australia (Jung et al., 2018). Several *Phythophtora* species (e.g., *Phytophthora cinnamoni*, *Phytophthora cryptogea*, *Phytophthora kernoviae*) are currently present in New Zealand native and plantation forests (Beever et al., 2009; Scott & Williams, 2014). Studies have shown that *P. cinnamomi* results in fine root death and symptoms of chlorosis in New Zealand native tree species such as *Agathis australis* and *Phyllocladus trichomanoides*, affects regeneration and thus may alter the long‐term vegetation dynamic of these forests (Horner, 1984; Johnston et al., 2003; Podger & Newhook, 1971).

Pathogen‐driven host dieback and mortality and subsequent changes in plant species composition are likely to affect biogeochemical fluxes. Given their rapid response to disturbance and environmental change, water‐bound ecosystem fluxes function as an early marker of shifting ecosystem conditions (Likens, 2013; McClain et al., 2003). Throughfall and stemflow are critical components of the hydrological and biogeochemical cycles of forest ecosystems, as they are the main pathways for transferring precipitation, solutes, organic matter, and microorganisms from the phyllosphere (aboveground surfaces of plants) to the pedosphere (Levia & Frost, 2006; Parker, 1983; Van Stan & Stubbins, 2018). Of particular importance in ecosystems’ internal element cycling is the transfer of plant nutrients. Inorganic nutrients are essential or beneficial to fundamental physiological processes of plant metabolism, plant growth and fitness, and soil fertility (Marschner, 2011; Parker, 1983; Schulze et al., 2019). Precipitation passing through the canopy as throughfall and flowing along stems as stemflow becomes enriched in micro‐ and macronutrients (Staelens et al., 2007). Another important ecosystem compartment in the internal nutrient cycle is the forest floor. Solution collected underneath the forest floor (forest floor leachate) is often greatly enriched in nutrients compared to bulk precipitation and canopy water fluxes due to the leaching of nutrients from organic matter (Batjes, 1996). Nutrient transfer to the soil by throughfall deposition and forest floor leachates is an important input pathway, particularly in ecosystems where nutrient return through litterfall decomposition is slow (van Langenhove et al., 2020; Will, 1959) and in ecosystems with low soil nutrient availability (Forti & Moreira‐Nordermann, 1991; Moslehi et al., 2019; Parker, 1983).

The magnitude of nutrient concentration and fluxes in throughfall, stemflow, and forest floor leachate are the result of the interaction of several variables, including meteorological conditions (Dunkerley, 2014), seasonality (Macinnis‐Ng et al., 2014), intra‐ and interspecies differences among tree species (Lilienfein & Wilcke, 2004; Schroth et al., 1999) and vegetation structure (Crockford & Richardson, 2000; Levia & Herwitz, 2002). Plant health (e.g., plant nutrient status, mechanical injury, insect pest, and pathogen‐related damage) has also been shown to affect canopy water yields and nutrient fluxes. For example, annual throughfall fluxes of Ca, Mg, and Fe were up to 100% higher beneath spruce trees affected by air pollutants, insects and microfungi in Sweden, while Mn and K fluxes decreased (up to 30%) compared to throughfall fluxes from healthy reference trees (Alenäs & Skärby, 1988). Bark beetle caused forest dieback in a Norway spruce‐dominated forest in the Czech Republic resulted in a decrease in K, Ca and Mg concentrations in throughfall (Kopáček et al., 2017). Defoliation caused by the pale tussock moth's larvae in a beech forest in southern Sweden resulted in an increase in K throughfall flux (Nilsson, 1978). Varying canopy nutrient flux responses to biotic disturbances may partly be driven by differences in the interaction of the pest and/or pathogen and host and the role of the host in the forest (Lovett et al., 2002). However, the implications of plant pathogen caused forest dieback, especially through fungus‐like organisms such as *Phytopththora* species, on canopy and forest floor nutrient fluxes remain largely unknown.

*Phytophthora agathidicida* is a novel and significant threat to the survival of the endemic, ecologically unique and culturally important kauri forests in the northern part of New Zealand. Kauri is endemic to northern New Zealand (north of latitude 38°S) (Ecroyd, 1982) and is the largest and longest‐living tree species in the country. A distinctive plant community is found underneath mature kauri trees (Wyse et al., 2014) which has been associated with the particular soil characteristics found in kauri‐dominated forests such as low pH and high ammonium concentrations (Verkaik et al., 2007). Undisturbed kauri stands are characterized by thick forest floors (litter plus organic layer) with mean residence time of 9–78 years (Silvester & Orchard, 1999). Throughfall nutrient fluxes (sum of Na, Ca, K, and Mg) were 2.5‐times higher than litterfall nutrient fluxes (Reed, 1984), suggesting that throughfall contributed significantly to the amount of nutrients reaching the pedosphere (Reed, 1984; Sangster, 1986).

In this study, we use canopy and forest floor nutrient data collected over one year to test whether canopy and forest floor plant nutrient fluxes change along a *P. agathidicida* infection gradient in a kauri‐dominated forest. In a previous study, using the same study design, we investigated the particulate and dissolved organic matter fluxes in this kauri‐dominated forest. Organic matter and inorganic nutrients differ considerably in their chemical composition, transformation mechanisms, and their role within ecosystems (Qualls et al., 2000).

## 2 MATERIAL AND METHODS

### 2.1 Study area

**FIGURE 1:**
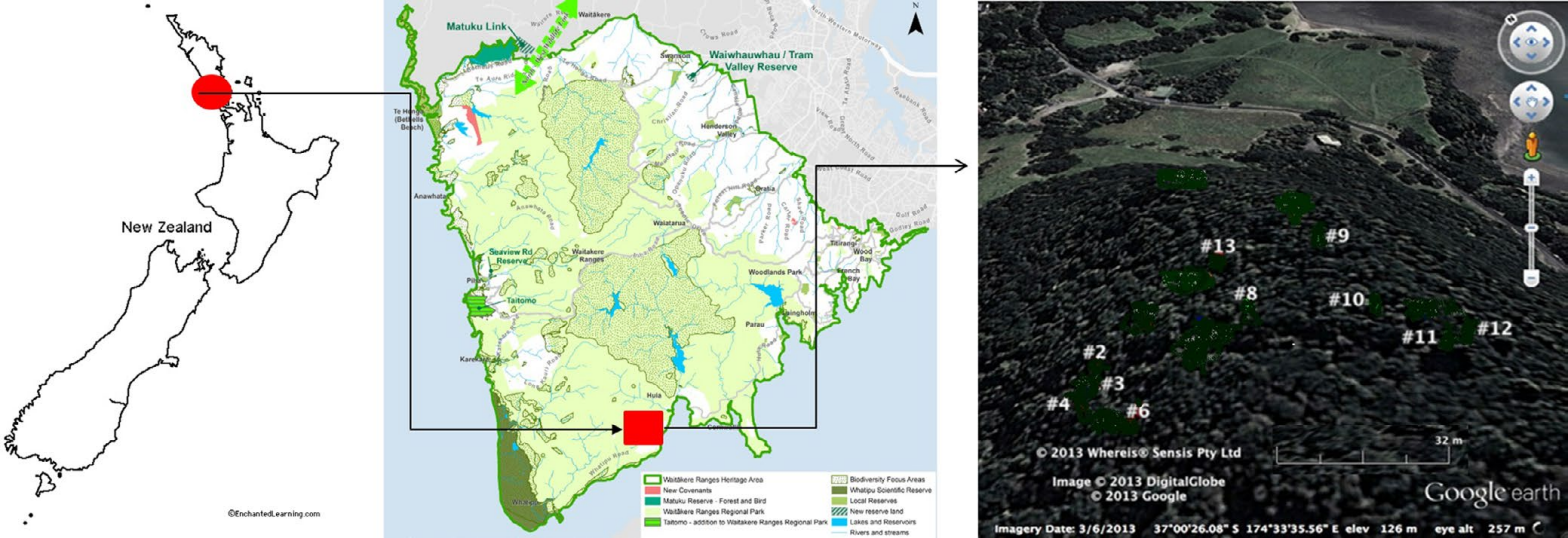
Map of study area and location of investigated kauri trees, Huia, Waitakere Ranges Regional Park, Auckland, New Zealand

This study was conducted in the Karamatura Valley (37°00’S, 174°33’E) near Huia. Huia is located ~ 30 km southwest of central Auckland, New Zealand, in the southern part of the Waitakere Ranges Regional Park. The study site (approximately 2,500 m^2^) is located along a northeast facing ridgeline between 125 and 140 m above mean sea level, with slopes ranging between 5º and 15º (Figure 1).

Mean annual temperature in the area is 14°C. Total annual rainfall measured at a nearby station (Arataki, 7.5 km northeast of the study area, 190 m above sea level) is approximately 1,600 mm (1981–2010) (Environmental Monitoring, Auckland Council GeoMaps, https://geomapspublic.aucklandcouncil.govt.nz/viewer/index.html). Approximately 70% of annual rainfall occurs between June and August (austral winter).

The soils are loams/sandy loams and classified as Typic Haplohumult (US Soil Taxonomy; Soil Survey Staff, 2014), and developed from andesitic grit, sand, and siltstone (Hayward, 1976). The parent material originates from volcanic conglomerate and lava flows caused by volcanic eruptions 12–25 million years ago (Searle & Mayhill, 1981). The area is characterized by a steep and rugged topography with elevation ranging from sea level to 475 m above sea level.

Regenerating native shrubland and forests, wetland, and dune ecosystems are the predominant vegetation types in the Waitakere Ranges. The area has been extensively logged in the mid‐late 19th and early 20th centuries (Esler, 1983). Kauri is the dominant canopy and subcanopy species covering over 70% of the study site. Other conifer tree species are *Phyllocladus trichomanoides* and *Dacrydium cupressinum*. Other common understory species are *Kunzea ericoides, Hedycarya arborea*, *Leionema nudum,* and *Cyathea dealbata*.

A change in the health status of kauri trees in the Waitakere Ranges Regional Park during the mid‐2010s and subsequent investigations have shown that *P. agathidicida* is the underlying agent of kauri dieback (Waipara et al., 2013; Weir et al., 2015). Recent studies (Hill & Waipara, 2017) estimated that approximately 30% of the kauri‐dominated forests in the Waitakere Ranges Regional Park exhibit symptoms of kauri dieback. Although kauri dieback in the area has been observed over the last 15 years, it is unknown for how long *P. agathidicida* has been present in this particular forest stand. It should also be noted that kauri dieback symptoms may be caused by other agents (e.g., other pests and/or pathogens) or environmental conditions (e.g., drought). Furthermore, kauri roots may already be infected but trees may not show any visual symptoms, as there may be a time lag between infection and the appearance of visual symptoms. Little is known about the incubation period, which may vary among kauri individuals and among environmental site conditions (Bradshaw et al., 2020).

### 2.2 Tree characteristics

Ten kauri trees differing in their health status were selected for this study. Tree health status was visually evaluated by examining crown and trunk conditions, including leaf yellowing, canopy thinning, and occurrence of resin bleeding (Hill & Waipara, 2017; Waipara et al., 2013).

The diameter of the selected kauri trees was measured at 1.3 m above the ground using a diameter tape (Table 1). Crown radii (= distance from the center of the trunk to the perimeter of the crown) in eight cardinal directions were measured in the field. The crown projection area was estimated using the mean of the crown radii (Pretzsch et al., 2015). The thickness of the forest floor (litter plus organic layer) was measured using a retractable tape.

**TABLE 1:** Tree characteristics, health status, and soil *P. agathidicida* DNA concentration

Tree number	Tree health status^a^(2015)	Basal area (2015)	Crown projection area (2013)	Canopy density (2013)	Median soil *P. agathidicida* DNA concentration (2015)	Forest floor (2015)
		m^2^	m^2^	%	fg/μL	cm
2	10	0.155	17.1	82.9	56.4	2.0
3	9	0.256	42.1	69.4	56.6	3.0
4	8	0.276	100.7	74.2	48.8	3.0
6	7	0.311	79.3	72.6	27.9	3.0
8	4	0.658	102.3	79.6	25.4	12.0
9	3	0.243	31.9	83.7	42.0	4.0
10	5	0.353	80.7	75.6	21.3	6.5
11	1	0.435	40.5	81.8	23.1	15.0
12	2	0.177	30.7	86.7	25.4	4.0
13	6	0.290	83.5	83.9	35.5	6.5

^a^ Visual assessment: Scale from 1 to 10 with 1 indicating “minimal visual signs of dieback” and 10 as “dead.”

Canopy density (also referred to as canopy closure) was determined from photographs taken at 1.5 m above the ground (next to the throughfall collectors) using a digital camera (OM‐D E‐M5, Olympus, Olympus 12–50 mm lens set at 12 mm) and the computer software CanopyDigi developed by Goodenough and Goodenough (2012).

To minimize the risks of spreading the pathogen, we followed the “Hygiene procedures for kauri dieback” (Kauri Dieback Management Programme, 2013) by cleaning and disinfecting (using SteriGENE) footwear upon entering and leaving the study site, and all equipment was sanitized between use on different trees.

### 2.3 Quantification of *P. agathidicida* DNA concentration in soil

Quantitative PCR (qPCR) was employed to detect *P. agathidicida,* and to quantify the *P. agathidicida* DNA concentration in the soil, using the TaqMan approach (Than et al., 2013). Mineral soil (0–10 cm) samples were taken at one meter distances to the trunk in four cardinal directions, and each sample was analyzed separately (Singh et al., 2017). The DNA was extracted from the soil using the PowerSoil®DNA Isolation Kit (MO BIO Laboratories Inc., Solana Beach, CA, USA). The ITS (internal transcribed spacer) region was amplified using a TaqMan probe and primers ITS_F2 and ITS_R3 (Than et al., 2013). The qPCR was performed on an ABI Prism 7900HT Sequence Detection System (Applied Biosystems) and then analyzed by the qPCR analysis software, SDSv2.4 (Applied Biosystems). The absolute quantification method was used to infer the DNA concentration of *P. agathidicida* in each of the samples. A standard curve was generated by plotting the log10 DNA concentration of standards, against their mean cycle threshold value, which was determined spectrophotometrically at the exponential phase of the qPCR process. A linear regression model was applied to estimate the *P. agathidicida* DNA concentration (fg/µL) in each soil sample. The values presented are the median soil *P. agathidicida* DNA concentration of the samples taken in four cardinal directions. Further details of the methodology are given in Singh et al., 2017).

### 2.4 Water sampling

**FIGURE 2:**
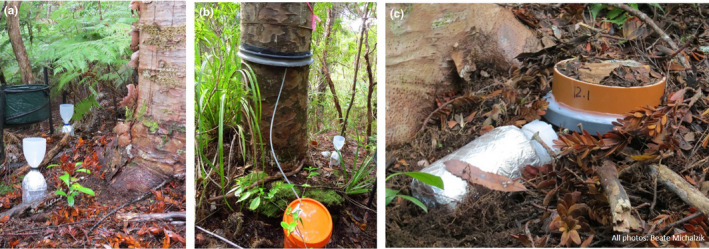
Canopy and forest floor leachate collectors: (a) throughfall, (b) stemflow, and (c) free‐draining lysimeter

Bulk precipitation collectors (*n* = 3, spaced 10 m apart) were located in a pasture (approximately 150 m from the study area, 50 m above sea level). The collectors consisted of plastic funnels (area = 113 cm^2^) mounted on top of 2 L polyethylene (PE) bottles. The same type of collector was used for throughfall (Figure 2a).

Throughfall collectors (*n* = 5 per tree) were positioned in a cross‐shaped grid around each tree trunk. Four collectors were placed in the four cardinal directions at 1.5 m of the trunk, and one collector close to the trunk to capture the characteristics of the core crown area, following the approach described in Schroth et al., (2001). To avoid contamination by animal and plant debris, a 2‐mm PE mesh filter was placed at the bottom of the funnels. The PE bottles were washed frequently with HCl to reduce the growth of microorganisms. To minimize exposure to ultraviolet radiation and to prevent algae growth between sample collections, the collectors were wrapped in aluminum foil.

The stemflow collar consisted of a butyl inner tube and foam tube (4 cm in diameter, cut in half). The foam tube was wrapped around the trunk at approximately 1.3 m above the ground and fixed to the stem with the inner tube to create a watertight seal. The collars were sloped around the tree and connected to a PE tube fitted at the lowest point to drain off the stemflow into a 20 L PE bucket (Figure 2b).

Free‐draining lysimeters (*n* = 2 per tree) were used to collect forest floor leachate. One lysimeter was located at a 1.5 m distance from the trunk, and one was close to the trunk. The lysimeters (area = 298 cm^2^) consisted of polypropylene (PP) plates covered with a 0.5 mm PE net, and were connected with a PE tube to 2 L PE bottles (Figure 2c).

Bulk precipitation, throughfall, stemflow, and forest floor leachate were collected over one year. From 5 July to 30 September 2015, the sampling interval was weekly to fortnightly. Due to logistical constrains, samples were collected on a monthly basis from 27 October 2015 to 20 June 2016. Throughfall from the individual collectors of a given tree was pooled to one volume‐weighted sample per tree per collection date. The solution from each individual lysimeter was pooled to one volume‐weighted sample per collection date. Water volume in each individual sampler on a given sampling date was measured with a graduated cylinder.

### 2.5 Chemical analysis

Water samples were filtered (GF 6, Whatman, GE Healthcare, Buckinghamshire, UK, pore size < 1 µm) on the day of collection and then frozen (−18°C). Potassium (K, 0.1 mg/L), calcium (Ca, 0.1 mg/L), magnesium (Mg, 0.02 mg/L), phosphorous (P, 0.1 mg/L), sulfur (S, 0.3 mg/L), sodium (Na, 0.1 mg/L), silicon (Si, 0.005 mg/L), iron (Fe, 0.01 mg/L), and manganese (Mn, 0.003 mg/L) concentrations were measured using an inductively coupled plasma optical emission spectrometer (ICP‐OES, Varian 725‐ES, Agilent Technologies Australia Pty Ltd, Mulgrave, Victoria, Australia). Values in brackets give the detection limit of a given element. Nutrient concentrations below the detection limit were arbitrarily set to half of that limit for further calculations. Phosphorous (P) concentrations were below the detection limit in most ecosystem components, and are not presented. Nutrient concentrations were not measured in samples taken on 21 July, 2 August, 26 August, and 9 September 2015.

### 2.6 Water yield and nutrient fluxes

Bulk precipitation, throughfall, and forest floor leachate water yield were estimated in mm units as follows:

(1) $$\begin{array}{c} \text{Bulk precipitation}\;\left(\text{BP}\right),\text{throughfall}\left(\text{TF}\right),\text{forest floor}\left(\text{FF}\right)\text{leachate}\left(\text{mm}\right)\\ \;=\frac{\text{BP},\text{TF},\text{and FF volume}\left(\text{BP},\text{TF},\;\text{FF}\right)\left(L\right)}{\text{collector area}\;\left(0.0113\;m^{2}\;\text{for}\;\text{BP and TF};\;0.0298\;m^{2}\;\text{F for FF}\right)} \end{array}$$

Stemflow yield was estimated by dividing the stemflow volume by the crown projection area of a given tree to obtain stemflow yield per tree (= stemflow depth) (Park & Cameron, 2008; van Stan & Stubbins, 2018).

(2) $$\text{Stemflow}\left(\text{mm}\right)=\frac{\text{stemflow}\;\text{volume}\left(L\right)}{\text{crown}\;\text{projection}\;\text{area}\left(m^{2}\right)}$$

Frequent overflow of stemflow collectors was observed for trees 3, 4, and 13. Thus, stemflow yield could not be calculated for these trees. In the case of missing stemflow values, or overflow of the remaining trees, gap filling was done. Regression analysis was applied to determine the relationship between bulk precipitation and stemflow, and throughfall and stemflow. These functions (linear or polynomial) were used to estimate stemflow volumes for collection dates with missing stemflow data or stemflow overflow.

The nutrient input (mg/m^2^ sampling date) to the mineral soil through the various water pathways for a given sampling date was calculated by multiplying nutrient concentrations (mg/L) with the corresponding water volume (mm). The nutrient flux of all collection sampling dates was summed together to obtain the annual nutrient yield per tree (mg/m^2^ year).

To estimate the nutrient flux for collection dates with missing nutrient concentrations, we determined the relationship between water yield and nutrient fluxes for a given tree across the sampling period using regression analysis.

To assess the canopy effect on bulk precipitation chemistry, the net throughfall nutrient fluxes were calculated as follows (Parker, 1983):

(3) $$\text{Net}\;\text{throughfall}\;{\text{flux}}_{x}=\text{throughfall}\;{\text{flux}}_{x}‐\text{bulk}\;\text{precipitation}\;{\text{flux}}_{x}$$

where x is a given macro‐, micro‐, or beneficial nutrient.

The contribution of seasalt‐derived elements was estimated as follows (Keene et al., 1986; Pierret et al., 2019):

(4) $$\text{Enrichment}\;\text{factor}\left(X\right)=\frac{{\left(\frac{X}{\text{Na}\;\text{or}\;\text{Mg}}\right)}_{\text{BP},\text{TF},\text{SF},\text{FF}}}{{\left(\frac{X}{Na\;or\;Mg}\right)}_{\mathrm{seawater}}}$$

where X/Na or Mg _BP, TF, SF, FF_ is the ratio between the element X and the concentration of Na or Mg in the corresponding sample of bulk precipitation, throughfall, stemflow, and forest floor leachate. X/Na or Mg_seawater_ is the ratio between the element X and the concentration of Na or Mg in seawater.

These calculations were based on the assumption that Na or Mg originated from sea spray, and had a conservative behavior. Na and Mg concentrations were strongly correlated (rho = 0.860, *p* <.001) suggesting that Mg in this ecosystem is mainly seasalt‐derived. An enrichment factor > 1 indicates a contribution of nonsea sources (Keene et al., 1986; Mimura et al., 2016; Pierret et al., 2019).

### 2.7 Statistical analysis

In view of the non‐normal distribution of water yields, nutrient concentrations, and fluxes, nonparametric statistical tests (Kruskal–Wallis followed by post‐hoc separation; significant at *p* <.05) were used to assess differences in median values between ecosystem components (bulk precipitation, throughfall, stemflow, and forest floor leachate). The relationships between soil *P. agathidicida* DNA concentration, tree characteristics (canopy density, forest floor depth), annual water yield, and nutrient fluxes were tested using Spearman rank correlation analysis.

Linear mixed‐effect models were used to identify the effect of soil *P. agathidicida* DNA concentration and canopy density on response variables (water flux, nutrient concentrations and fluxes) in throughfall, stemflow, and forest floor leachate. Visual tree health status (*rho* = 0.723, *p* =.018, *n* = 10) and tree diameter (*rho* = −0.650, *p* =.042, *n* = 10) were significantly correlated with soil *P. agathidicida* DNA concentration and thus were not included in the linear mixed‐effect models. Soil *P. agathidicida* DNA concentration (expressed as a z‐score), canopy density (expressed as a z‐score), water flux (when testing nutrient concentrations), and forest floor depth (when testing nutrient concentration and fluxes in forest floor leachate) were defined as fixed effects. Sampling date (expressed as days since the start of sample collection) was included as a random factor to account for repeated measures. Simple (simple effects) models included soil *P. agathidicida* DNA concentration, canopy density, water flux (when testing nutrient concentrations), forest floor depth, and sampling date. Complex models contained all factors without interactions, and maximal models contained all factors and interactions. In cases of nonhomogeneity of residuals, the response variables were transformed. The models were run using restricted maximum‐likelihood estimation. The Akaike's information criterion (AIC) value was calculated for each linear mixed model. The difference in AIC between models was calculated (AIC_i_‐AIC_min_). The models with the highest explanatory power were identified when the model with the lowest AIC value differed by more than three from other models (Burnham & Anderson, 2002).

All statistical analyses were conducted using the software package IPM SPSS Statistics version 25 (IBM Corporation, Chicago, IL, USA).

## 3 RESULTS

### 3.1 Nutrient concentrations and fluxes in bulk precipitation, throughfall, stemflow, and forest floor leachate

Median concentrations in bulk precipitation varied by several orders of magnitude (ppb to ppm) between elements in the order Na > S > Mg > K > Ca > Si >Mn > Fe (Table 2). Significant differences in nutrient concentrations were only found between bulk precipitation and stemflow, and between bulk precipitation and forest floor leachate (Table 2). We found a clear seasalt contribution for Na in throughfall, stemflow, and forest floor solutions, and for S in all components with enrichment factors between 0.44 and 0.86. Magnesium originated partly from seasalt with enrichment factors between 0.69 and 0.89 for bulk precipitation, throughfall, and stemflow.

**TABLE 2:** Macro‐ (K, Ca, Mg, S), micro‐ (Fe, Mn), and beneficial (Na, Si) nutrient concentrations in bulk precipitation, throughfall, stemflow, and forest floor leachate, Huia, New Zealand. Values are median, minimum (min), and maximum (max) concentrations across all trees (*n* = 10) and sampling dates (*n* = 17)

	K	Ca	Mg	S	Fe	Mn	Na	Si
	mg/L							
*Bulk precipitation*								
Median	0.591^a^	0.491^a^	0.841^a^	1.137^a^	0.0005^a^	0.0015^a^	5.558^a^	0.017^a^
Min.–max.	0.050–0.681	0.075–0.824	0.164–1.554	0.150–1.876	0.0005–0.0005	0.0015–0.007	1.461–10.110	0.008–0.281
*Throughfall*								
Median	6.715^a,b^	1.896^a^	2.900^a,b^	3.006^a,b^	0.0005^a^	0.024^a,b^	13.480^a,b^	0.035^a,b^
Min.–max.	0.218–23.840	0.178–6.658	0.257–13.160	0.150–11.580	0.0005–0.035	0.0015–0.245	1.461–62.930	0.005–0.112
*Stemflow*								
Median	10.040^b^	2.190^a^	3.221^b^	3.452^b^	0.012^b^	0.074^b^	15.840^b^	0.053^b^
Min.–max.	0.391–44.900	0.209–17.120	0.194–19.930	0.472–13.790	0.0005–0.078	0.002–0.316	1.767–66.230	0.008–0.197
*Forest floor leachate*								
Median	14.590^b,c^	8.478^b^	5.289^b,c^	4.993^b,c^	0.240^b^	0.046^b^	20.035^b^	0.161^b,c^
Min.–max.	2.465–51.940	1.517–27.940	0.782–15.620	0.385–12.460	0.089–0.809	0.002–0.780	1.645–65.090	0.039–0.683

Different letters indicate significant differences between water pathways for a given element.

Medians of annual water flux decreased as precipitation percolated through the canopy and forest floor: bulk precipitation > throughfall > forest floor leachate (Table 3). Nutrient fluxes in throughfall were higher than in bulk precipitation (Table 3). Normalized to the crown projection area, stemflow nutrient fluxes contributed less than 3% of annual bulk precipitation nutrient fluxes. Forest floor leachate was enriched in all nutrients compared to bulk precipitation and stemflow, and had significantly higher Ca, Fe, Si fluxes than throughfall (Table 3).

**TABLE 3:** Annual macro‐ (K, Ca, Mg, S), micro‐ (Fe, Mn), and beneficial (Na, Si) nutrient fluxes in bulk precipitation, throughfall, stemflow, and forest floor leachate, Huia, New Zealand. Values are median, minimum (min), and maximum (max) fluxes across all trees (*n* = 10 for throughfall, *n* = 10 for forest floor leachate, *n* = 7 for stemflow). Different letters indicate significant differences between water pathways for a given element

	Water yield	K	Ca	Mg	S	Fe	Mn	Na	Si
	mm/year	mg/m^2^ year							
*Bulk precipitation*									
Median	1693	1,000.56^a^	717.83^a^	1,692.15^a^	1,924.94^a^	0.85^a^	4.23^a^	9,409.69^a^	28.49^a^
*Throughfall*									
Median	1,125	9,135.17^b^	2,277.70^b^	3,988.40^b^	3,521.08^b^	2.67^b^	32.24^b^	17,091.16^b^	33.23^a^
Min	719	5,830.03	1,508.78	1,959.32	1,844.03	1.98	20.18	8,759.80	27.23
Max	1,319	12,947.64	3,140.06	5,387.15	5,071.22	6.11	61.77	22,542.41	45.62
*Stemflow*									
Median	1.433	14.37^c^	4.93^c^	4.66^c^	5.26^c^	0.02^c^	0.11^c^	27.86^c^	0.10^b^
Min	0.424	4.24	0.85	1.12	1.23	0.01	0.03	6.06	0.02
Max	2.537	34.12	6.60	11.33	10.41	0.03	0.26	51.78	0.12
*Forest floor leachate*									
Median	793	12,876.50^b^	6,966.98^d^	4,737.33^b^	4,361.42^b^	220.04^d^	53.16^b^	15,464.25^b^	141.99^c^
Min	709	9,910.17	4,567.42	3,378.62	3,045.18	137.13	30.49	11,531.06	108.22
Max	962	21,407.08	8,201.97	7,718.33	6,740.08	320.66	82.15	23,476.63	249.14
*Net throughfall flux*									
Median		8,134.61	1,559.87	2,296.25	1,596.14	1.82	28.01	7,681.47	4.75
Min		4,829.47	790.94	267.16	−80.91	1.13	15.95	−649.89	−1.25
Max		11,947.07	2,422.91	3,694.99	3,146.28	5.26	57.54	13,132.72	17.13

### 3.2 Effect of tree, soil, and infection metrics on nutrient concentrations and fluxes

We found significant negative correlations between soil *P. agathidicida* DNA concentrations and throughfall Ca (*rho* = −0.723, *p* =.018), K (*rho* = −0.833, *p* =.003), Mg (*rho* = −0.717, *p* =.02), and S (*rho* = −0.857, *p* =.002) concentrations, but no correlation between throughfall nutrient concentrations and canopy density. Stemflow nutrient concentrations were neither significantly correlated with soil *P. agathidicida* DNA concentration nor with canopy density. In contrast, forest floor leachate Mg (*rho* = −0.644, *p* =.044), Na (*rho* = −0.687, *p* =.028), S (*rho* = −0.657, *p* =.039), and Si (*rho* = −0.705, *p* =.023) concentrations were negatively correlated with soil *P. agathidicida* DNA concentration, and forest floor leachate. Na (*rho* = 0.753, *p* =.012) and Si (*rho* = 0.833, *p* =.003) concentrations were positively correlated with forest floor depth.

**FIGURE 3:**
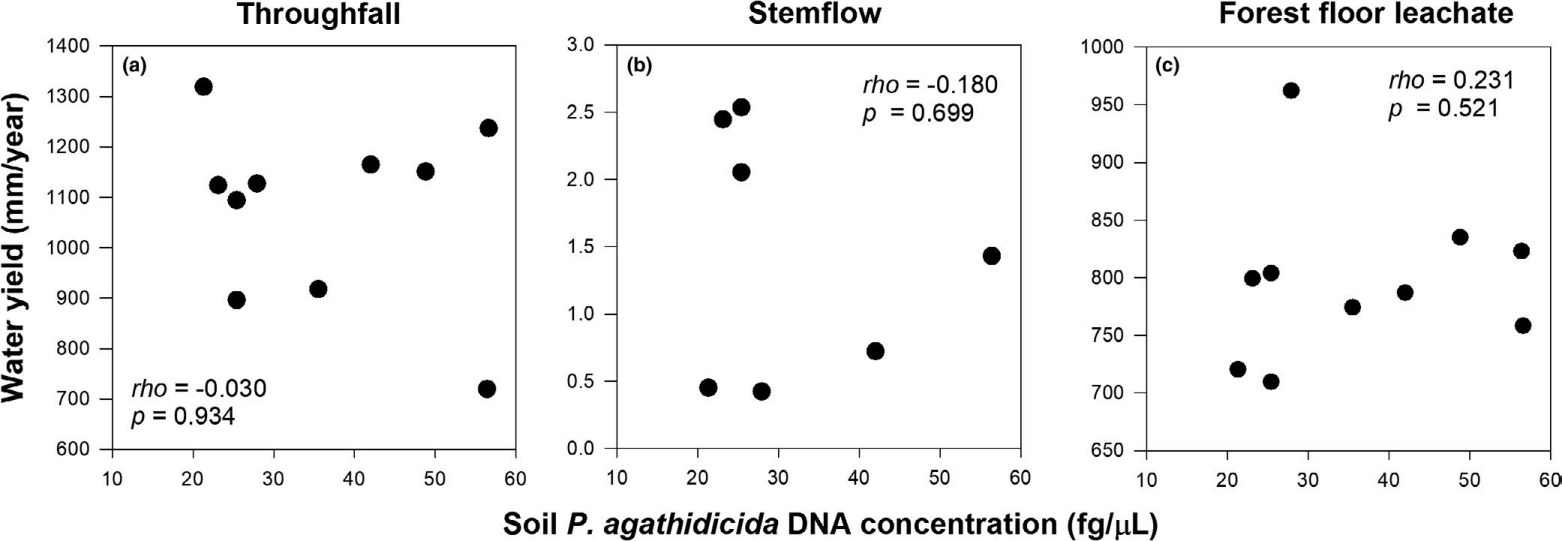
Soil *P. agathidicida* DNA concentration versus annual water yield of (a) throughfall, (b) stemflow, and (c) forest floor leachate

**FIGURE 4:**
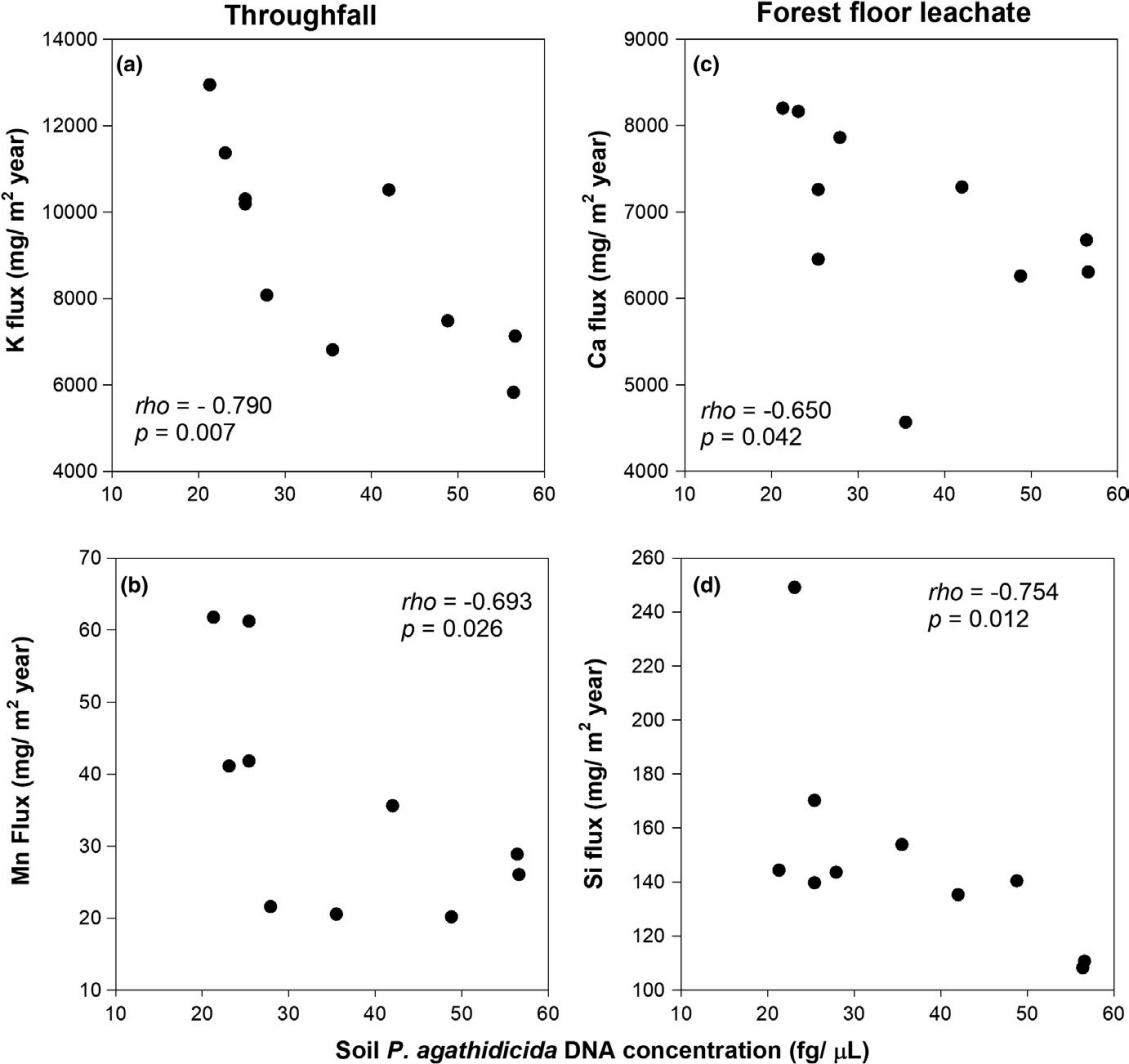
Soil *P. agathidicida* DNA concentration versus annual throughfall (a) potassium and (b) manganese fluxes and annual forest floor (c) calcium and (d) silicon fluxes

No significant correlation was found between throughfall, stemflow, and forest floor leachate water yield and soil *P. agathidicida* DNA concentration (Figure 3a‐c). Throughfall K and Mn fluxes and forest floor Ca and Si fluxes were significantly negatively correlated with soil *P. agathidicida* DNA concentrations (Figure 4a‐d). A positive correlation was found between forest floor depth and forest floor Mn (*rho* = 0.704, *p* =.023) and Si (*rho* = 0.642, *p* =.045) fluxes.

Linear mixed‐effect models at tree level enabled separating the effects of canopy density, forest floor depth and soil *P. agathidicida* DNA concentration on nutrient concentration and fluxes. The results revealed that soil *P. agathidicida* DNA concentration significantly affected throughfall S, K, Ca, Mg, and Mn fluxes (Table S1b) and net throughfall fluxes of Ca, K, and Mg (Table S2). No effect of soil *P. agathidicida* DNA concentration was found for stemflow nutrient concentrations and fluxes (Table S3a, b). Linear mixed models revealed a significant effect of soil *P. agathidicida* DNA concentration on K, Mg, and Mn concentration in forest floor leachate (Table S4a).

## 4 DISCUSSION

### 4.1 Internal transfer of nutrients

The proximity of the study area to the coast (Manukau Harbour and Tasman Sea) partly explains that a considerable proportion of Na, S, and Mg in bulk precipitation and throughfall originates from marine sources. A close association between S, Na, Mg, and K in bulk precipitation has been reported at stations located near the ocean, independent of longitude and latitude (Galloway et al., 1982; Ledgard & Upsdell, 1991; McDowell et al., 1990; Pierret et al., 2019). Seasalt influencing throughfall chemistry has been observed across a range of conifer and broadleaf forests located near the ocean (Farrell et al., 1998; Pierret et al., 2019; Westman, 1978).

Higher nutrient fluxes in throughfall compared to bulk precipitation as observed at Huia (Table 3) have also been reported across deciduous and evergreen forests (Carlisle et al., 1966; Parker, 1983; Staelens et al., 2007). However, the level of enrichment varied between nutrients (K (+ 800%) > Mn (+ 660%) > Ca (+ 217%) ≈ Fe (+ 215%) > Mg (+ 136%) > S (+ 83%) ≈ Na (+ 82%) > Si (+ 17%)). These differences are likely driven by nutrient availability and leachability. The cycling rate of K is fast, compared to other elements (Cole & Rapp, 1981), and as a monovalent cation, K is the most mobile element in the canopy and is readily leached (Parker, 1983; Staelens et al., 2003). In contrast, divalent cations (e.g., Ca, Mg) are more strongly bound (Cape, 1993; Pallardy, 2007). Magnesium and Fe occur in plants in chelated form and are less easy to leach, although Mn exhibits the second‐highest increase among the studied nutrients, which might point to pathogen‐induced leaf tissue damages or early leaf senescence.

Our throughfall sampling design (placing collectors exclusively under tree crowns) may have resulted in throughfall nutrient concentrations and fluxes biased high compared to the random placement of collectors across the forest stand. However, we argue that a comparison between macro‐, micro‐, and beneficial nutrients and nutrient fluxes across ecosystem compartments is still valid to gain insight into the internal nutrient cycling in this kauri‐dominated stand and to provide background information to assess the implications of kauri dieback on the fate and transport of nutrients at the ecosystem level.

The contribution of stemflow to ecosystem nutrient fluxes at Huia was small which can be partly explained by the low annual stemflow water yield (1.43 mm/yr; 0.8% of bulk precipitation) (Table 3). Stemflow normalized to the crown projection area accounted for 1.0%–4.7% of bulk precipitation in kauri‐dominated forests New Zealand (Fowler, 2015; Sangster, 1986). A global review showed that stemflow in two‐thirds of the studies was less than 2% of bulk precipitation (van Stan & Gordon, 2018). However, stemflow can be enriched in nutrients (10–100 times higher than bulk precipitation and throughfall) and thus be an important spatially localized nutrient input to the soil near tree stems (Enright, 1987; van Stan & Gordon, 2018).

Forest floor nutrient fluxes in this kauri‐dominated forest were higher (Fe (+ 8,082%) > Si (+ 326%) > Ca (+ 205%) > Mn (+ 64%) > K (+ 38%) > S (+ 24%) > Mg (+ 19%)) than bulk precipitation nutrient fluxes. The high Fe flux was astonishing, given that Fe content in leaves and plant biomass is low (< 100 μg/g DW) (Schulze et al., 2019). We suggest that Fe might be derived from the dissolution of ferric oxides by water‐soluble organic compounds (carboxylic acids, polyphenols) leached from kauri leaves and organic matter (Bloomfield, 1955). We assume that the release of the other nutrients from the forest floor is due to leaf litter decomposition and leaching. The nutrient concentrations in dead kauri leaves decline in the order Ca > K > Na = Mg (Enright & Ogden, 1987), which roughly aligns with the sequence given above.

To assess the input of nutrients reaching the soil by throughfall and forest floor leachates versus litterfall, we compared throughfall and forest floor leachate nutrient fluxes at Huia (Table 3) with annual litterfall nutrient fluxes measured in a nearby kauri‐dominated forest at Huapai (Enright, 2001). Forest structure, basal area, and litterfall at Huia (van der Westhuizen, 2014) and Huapai (Enright, 2001) are similar. The amount of K and Mg returned to the forest floor by throughfall was 2 and 2.5 times higher, respectively, than by litterfall. The leaching of K and Mg from the forest floor was up to three times larger than K and Mg input via litterfall. This is in line with results from a study conducted at Huapai where nutrient input via throughfall and stemflow was three times that from litterfall (Reed, 1984). Large buildup of organic matter (up to 2 m close to tree trunks) with resident times of up to 78 years (Silvester & Orchard, 1999) partly explains the lower nutrient return through litterfall. Similar findings have been reported from exotic conifer stands in New Zealand where throughfall reaching the soil under Douglas fir contained twice as much K as litterfall (Will, 1959), while atmospheric deposition plus canopy leaching delivered three times more K to the soil than the internal nutrient recycling via litterfall (van Langenhove et al., 2020). Nutrient input by throughfall and forest floor leachates may be critical in sustaining forest productivity, particularly in ecosystems characterized by low soil nutrient availability, such as kauri forests (Steward & Beveridge, 2010; Verkaik et al., 2006).

### 4.2 Effect of *P. agathidicida* infection on nutrient concentrations and fluxes

#### 4.2.1 Throughfall

With increasing soil *P. agathidicida* DNA concentration, throughfall nutrient concentration and fluxes decreased (Figure 4a‐d). The same pattern has been found for dissolved N and C concentration and fluxes (Schwendenmann & Michalzik, 2019). High variability in water yield (700–1350 mm; Figure 3a) may partly explain the lack of a relationship between soil *P. agathidicida* DNA concentration and water yield (Schwendenmann & Michalzik, 2019). Further, differences in canopy density between trees (Table 1) did not seem to be large enough to affect throughfall water yield.

**FIGURE 5:**
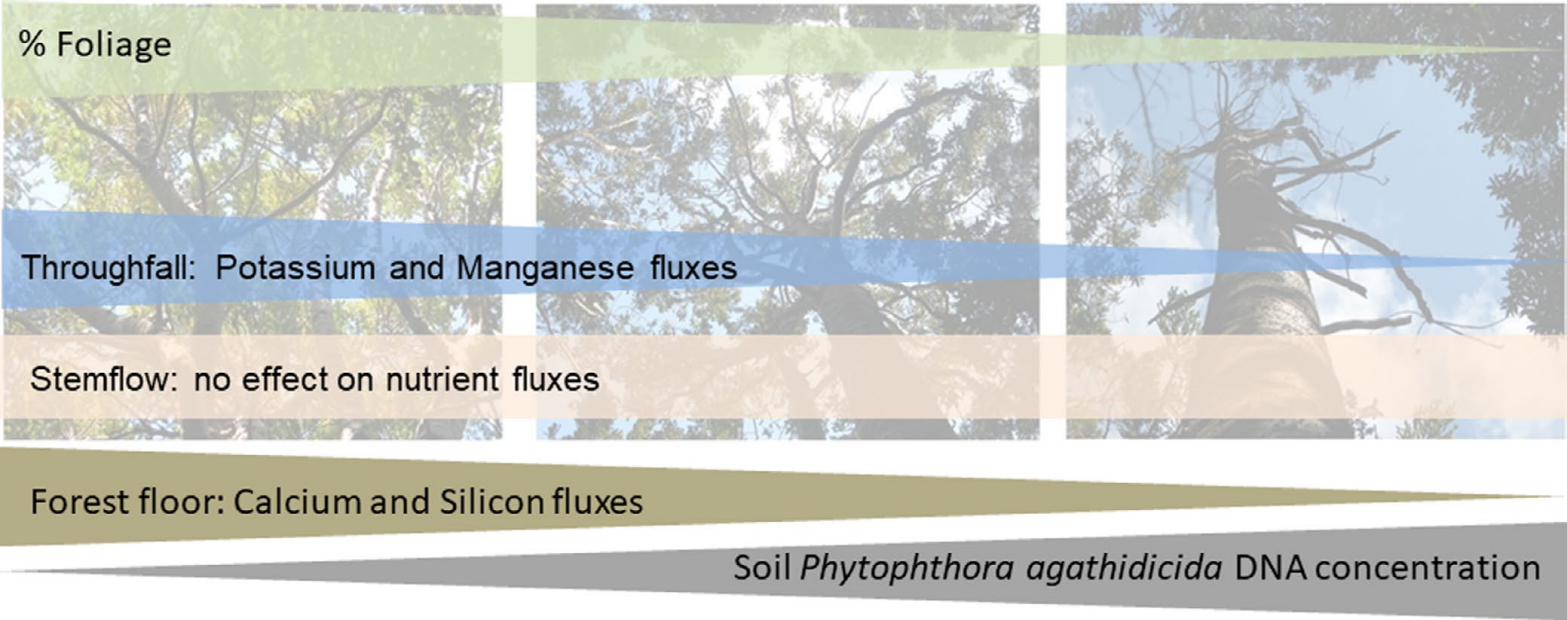
Effect of increasing soil *P. agathidicida* DNA concentration and decreasing foliage cover on nutrient fluxes

The following processes may explain the observed decline in nutrient concentration and fluxes in throughfall:

1. Decline in leaf nutrient concentration, which reduced the amount of nutrients leached from foliage. Like many other *Phytophthora* species, *P. agathidicida* infects the roots. Fine roots become necrotized; then, the pathogen may progress up the root and into the root crown, or invade xylem vessels (Jung et al., 2018). Root dieback and/or the obstruction of nutrient and water transport via the xylem limits the uptake of nutrients. The visible symptoms are often chlorosis and wilting of leaves, a thinning of the crown, and eventually the death of the infected tree (Oßwald et al., 2014). Studies reported lower leaf N concentrations in infected *Quercus robur* (Jönsson, 2004; Jönsson et al., 2003) and *Fagus sylvatica* seedlings (Fleischmann et al., 2004), which suggests a reduction in nutrient uptake, and/or translocation of nutrients from the leaves to the roots to sustain root production and/or produce defensive compounds (Jönsson, 2006). A ~ 30% reduction in Mg leaf content was observed in *Fagus sylvatica* seedlings infected with *Phytophthora citricola* (Wang et al., 2003). In contrast, substantial root dieback of *Quercus robur* seedlings following *Phytophthora quercina* and *Phytophthora cactorum* infection did not have a significant effect on leaf nutrient concentrations (Ca, Mg, K, Mn, Fe, S) (Jönsson, 2004).
2. Loss of foliage due to pathogen infection diminished the interaction of water with foliar surfaces, thus decreasing throughfall nutrient concentrations (Parker, 1983). Trees with higher soil *P. agathidicida* DNA concentration showed considerable canopy thinning (Figure 5), reducing washoff (McDowell & Likens, 1988; Parker, 1990), and foliar leaching (of potentially nutrient‐depleted foliage) (Czech & Kappen, 1997; Tukey, 1970).
3. Foliicolous lichens were found on some leaves shed by kauri trees characterized by higher soil *P. agathidicida* DNA concentrations. In a blue oak woodland in California, trees with epiphytic lichens had higher N, Ca, Mg, Na, and Cl depositions under their canopy than trees were lichens were removed (Knops et al., 1996). Based on this finding, we speculate that the loss of foliicolous lichens may partly contribute to the decrease in nutrient deposition.

#### 4.2.2 Stemflow

Soil *P. agathidicida* DNA concentration had no significant effect on stemflow nutrient concentrations and fluxes. This may partly be explained by the high variability in stemflow nutrient concentrations and water yield between trees masking the effect of *P. agathidicida* infection. Given that stems of highly infected trees showed a higher cover of vines, bryophytes (moss) and lichens (Fig. S1), we were expecting a change in stemflow nutrient concentrations and fluxes. Previous studies have shown that epiphytic lichens and bryophytes affect stemflow chemistry by selective uptake or release of nutrients (Jordan et al., 1980; Lang et al., 1976). For example, the depletion of stemflow N, P, and Ca fluxes in a subtropical montane forest has been attributed to nutrient uptake by the epiphytic bryophytes (Liu et al., 2002).

#### 4.2.3 Forest floor leachate

Forest floor leachate concentrations (Mg, Na, S, and Si) and fluxes (Ca, Si; Figure 4c,d) decreased significantly with increasing soil *P. agathidicida* DNA concentration.

The observed decrease in nutrients can partly be explained by the following processes:

1. Higher plant nutrient uptake due to changes in density and composition of understory plant communities. Understory plant density was higher underneath kauri trees characterized by high soil *P. agathidicida* DNA concentrations and severe canopy dieback. Studies have shown that dieback or removal of dominant trees can lead to greater productivity of the remaining species (Klein & Perkins, 1987) and higher plant nutrient uptake. Understory plant species did not show any signs of dieback and are functionally different to kauri and kauri‐associated plant species (van der Westhuizen, 2014; Wyse et al., 2014). Several kauri‐associated species belong to the family of *Ericaceae* (Wyse et al., 2014). The replacement of these often slow‐growing “stress tolerant” plant species (Grime, 1977) by species with higher nutrient requirements may have resulted in an enhanced uptake of nutrients from the forest floor.
2. Lower forest floor nutrient reservoirs due to lower leaf litter nutrient concentrations, decrease in litterfall and decline in forest floor depth. Lower fine root N, P, Ca, Mg, and Fe content have been found in European beech seedlings with *P. citricola* (Wang et al., 2003). Differences in microbial and fungal communities between asymptomatic and symptomatic mature kauri trees (Byers et al., 2020) and changes in microclimatic conditions (van der Westhuizen, 2014) are likely to affect nutrient cycling.

### 4.3 Long‐term implications of a decline in canopy‐ and forest floor‐derived nutrient fluxes

Given that throughfall and forest floor leachate are an important pathway of plant‐available nutrients to the soil in this kauri‐dominated stand, the significant reduction in throughfall K, Mn and in forest floor Ca and Si fluxes may have long‐term implications on ecosystem processes, tree health, and host pathogen interactions (Oliva et al., 2014). Potassium plays a critical role in biochemical and physiological processes such as enzyme activation, amino acid synthesis, and carbohydrate metabolism, influencing plant growth and susceptibility of host plants to pathogens and insects (Amtmann et al., 2008; Dordas, 2008; Marschner, 2012). Manganese is critical for overall plant vigor and an important regulator of plant responses to stress and disease resistance (Dordas, 2008). Manganese inhibits (1) aminopeptidase production, reducing the supply of essential amino acids for fungal growth and (2) pectin methylesterase, a fungal enzyme that degrades host cell walls (Dordas, 2008; Marschner, 2012). Some studies have shown that Si can restrict fungal hyphae penetration by creating a physical barrier, or by producing antifungal compounds (Dordas, 2008). Further, Si has been identified as a beneficial nutrient for many vascular plant species, in particular under stress conditions (Pontigo et al., 2015). Calcium ions preserve the structural integrity and functionality of membranes and cell walls, increase host resistance to invasion, and inhibit mycelial growth and zoospore release (Sugimoto et al., 2010). Calcium applications have been shown to reduce Phytophthora root rot in citrus, avocado, and oak (Campanella et al., 2002; Messenger et al., 2000; Serrano et al., 2013).

Root or bark infection by Phytophthora species results in root dieback, which affects nutrient and water uptake (Oßwald et al., 2014). Root dieback hampering nutrient uptake, plus the lack of plant‐available nutrients in the soil, may accelerate the decline in tree health. Further, repairing infected tissues and/or building up plant defenses increases the carbon and nutrient requirement of the host (Olivia et al., 2014). Leaf nutrient deficiency and leaf loss induced weakening of plant defenses may facilitate the buildup of pathogen inoculum and produce further damages (Olivia et al., 2014). In summary, a long‐term reduction in plant‐available K, Mn, Si, and Ca may accelerate the decline in tree health and increase the susceptibility of kauri to *P. agathidicida* infection.

## CONFLICT OF INTEREST

The authors declare that there is no conflict of interest.

## AUTHOR CONTRIBUTION

**Luitgard Schwendenmann:** Conceptualization (equal); Data curation (equal); Formal analysis (equal); Funding acquisition (equal); Investigation (equal); Methodology (equal); Project administration (equal); Resources (equal); Validation (equal); Visualization (equal); Writing‐original draft (equal); Writing‐review & editing (equal). **Beate Michalzik:** Conceptualization (equal); Data curation (equal); Formal analysis (equal); Funding acquisition (equal); Investigation (equal); Methodology (equal); Project administration (equal); Validation (equal); Visualization (equal); Writing‐original draft (equal); Writing‐review & editing (equal).

## Supporting information

Fig S1

Tables S1‐S4

## Data Availability

The data that support the findings of this study are openly available in figshare at https://doi.org/10.17608/k6.auckland.13632380.v1
