# Supplementary figures and images for: Impact of Phytophthora agathidicida infection on canopy and forest floor plant nutrient concentrations and fluxes in a kauri‐dominated forest
Source: Ecol Evol. 2021 Mar 19;11(9):4310–24. doi: 10.1002/ece3.7326 (PMC8093678; doi:10.1002/ece3.7326)

**
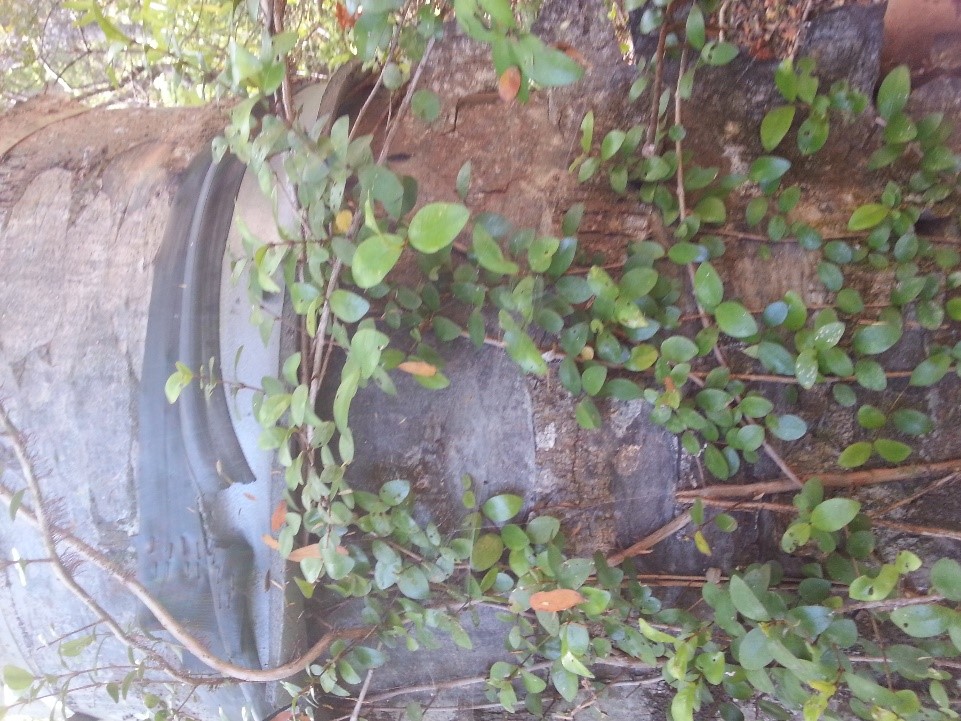
**

**Fig. S1** Kauri stem covered by vines

Supplement: Supplementary file 1 — Fig S1 [file ECE3-11-4310-s002.docx]
