# Supplementary material for: Impact of Phytophthora agathidicida infection on canopy and forest floor plant nutrient concentrations and fluxes in a kauri‐dominated forest
Source: Ecol Evol. 2021 Mar 19;11(9):4310–24. doi: 10.1002/ece3.7326 (PMC8093678; doi:10.1002/ece3.7326)
Supplement: Supplementary file 2 — Tables S1‐S4 [file ECE3-11-4310-s001.docx]

**Appendix**

Table S1-4. Estimated fixed effects of soil *Phytophthora agathidicida* DNA concentration (DNA), canopy density (CD), water yield (WY) and interactions using linear mixed models. Sampling date (23 June 2015 to 20 June 2016, n =18) was used as a random factor. The model/s with the highest explanatory power was identified when the model with the lowest Akaike Information Criterion (AIC) value differed by more than 3 from other models (Burnham and Anderson 2002).

Table S1a. Throughfall – Concentration (mg l^-1^)

|  | K concentration^1^ | | | | Ca concentration^1^ | | | | Mg concentration^1^ | | | | S concentration^1^ | | | |
| --- | --- | --- | --- | --- | --- | --- | --- | --- | --- | --- | --- | --- | --- | --- | --- | --- |
|  | Estimate | SE | p | AIC | Estimate | SE | p | AIC | Estimate | SE | p | AIC | Estimate | SE | p | AIC |
| Simple models |  |  |  |  |  |  |  |  |  |  |  |  |  |  |  |  |
| DNA | -0.162 | 0.047 | 0.001 | 217.179 | -0.197 | 0.051 | <0.0001 | 316.353 | -0.123 | 0.0431 | 0.006 | 222.319 | -0.117 | 0.0375 | 0.003 | 204.468 |
| Intercept | 2.110 | 0.454 | <0.0001 |  | 1.586 | 0.0489 | <0.0001 |  | 0.921 | 0.041 | <0.0001 |  | 0.954 | 0.0356 | <0.0001 |  |
| CD | 0.1596 | 0.0539 | 0.005 | 215.510 | 0.0932 | 0.0597 | 0.166 | 168.532 | 0.067 | 0.067 | 0.348 | 226.213 | 0.078 | 0.0402 | 0.059 | 209.683 |
| Intercept | 1.855 | 0.0507 | <0.0001 |  | 0.3835 | 0.0563 | <0.0001 |  | 0.841 | 0.063 | <0.0001 |  | 0.9661 | 0.0375 | <0.0001 |  |
| WY | -0.0048 | 0.0011 | <0.0001 | 227.935 | -0.0105 | 0.0008 | <0.0001 | 159.170 | -0.0114 | 0.0009 | <0.0001 | 193.700 | -0.0096 | 0.0008 | <0.0001 | 175.125 |
| Intercept | 2.008 | 0.0662 | <0.0001 |  | 1.529 | 0.0769 | <0.0001 |  | 2.1248 | 0.1059 | <0.0001 |  | 1.877 | 0.0808 | <0.0001 |  |
|  |  |  |  |  |  |  |  |  |  |  |  |  |  |  |  |  |
| Complex model |  |  |  | 228.996 |  |  |  | 162.863 |  |  |  | 195.121 |  |  |  | 178.137 |
| DNA | -0.1450 | 0.477 | 0.003 |  | -0.1202 | 0.0557 | 0.073 |  | -0.1887 | 0.0839 | 0.062 |  | -0.1395 | 0.5981 | 0.055 |  |
| CD | 0.0987 | 0.048 | 0.040 |  | -0.0183 | 0.0561 | 0.755 |  | -0.0889 | 0.0837 | 0.326 |  | -0.0165 | 0.0599 | 0.791 |  |
| WY | -0.0009 | 0.001 | 0.378 |  | -0.0108 | 0.0008 | <0.0001 |  | -0.0116 | 0.0009 | <0.0001 |  | -0.0096 | 0.0008 | <0.0001 |  |
| Intercept | 2.1933 | 0.0908 | <0.001 |  | 1.5196 | 0.0718 | <0.0001 |  | 2.111 | 0.994 | <0.0001 |  | 1.8415 | 0.7354 | <0.0001 |  |
|  |  |  |  |  |  |  |  |  |  |  |  |  |  |  |  |  |
| Maximal model |  |  |  | 225.817 |  |  |  |  |  |  |  |  |  |  |  | 201.333 |
| DNA | 0.1449 | 0.1135 | 0.205 |  | 0.0127 | 0.0796 | 0.873 | 185.905 | -0.0490 | 0.0854 | 0.573 | 237.423 | -0.0179 | 0.0765 | 0.819 |  |
| CD | -0.0936 | 0.1226 | 0.447 |  | 0.0641 | 0.0887 | 0.479 |  | 0.02473 | 0.0934 | 0.794 |  | 0.0218 | 0.0841 | 0.799 |  |
| WY | -0.0055 | 0.0012 | <0.0001 |  | -0.0125 | 0.0008 | <0.001 |  | -0.0146 | 0.0006 | 0.000 |  | -0.0115 | 0.0008 | <0.0001 |  |
| DNA × CD | 0.1567 | 0.1192 | 0.192 |  | 0.0955 | 0.0857 | 0.284 |  | 0.0514 | 0.0910 | 0.578 |  | 0.0657 | 0.0820 | 0.435 |  |
| DNA × WY | -0.0034 | 0.0014 | 0.015 |  | -0.0034 | 0.0009 | 0.001 |  | -0.0040 | 0.0008 | <0.0001 |  | -0.0036 | 0.0010 | 0.001 |  |
| DNA × CD × WY | -0.0024 | 0.0013 | 0.080 |  | -0.0027 | 0.0009 | 0.006 |  | -0.0034 | 0.0008 | <0.0001 |  | -0.0032 | 0.0010 | 0.002 |  |
| Intercept | 3.0892 | 0.1059 | <0.0001 |  | 1.6076 | 0.0717 | <0.0001 |  | 2.2115 | 0.7695 | <0.0001 |  | 1.9061 | 0.0691 | <0.0001 |  |

^1^response variable was ln transformed

Table S1a. Throughfall – Concentration (mg l^-1^)

|  | Fe concentration^1^ | | | | Mn concentration^1^ | | | | Na concentration^1^ | | | | Si concentration^1^ | | | |
| --- | --- | --- | --- | --- | --- | --- | --- | --- | --- | --- | --- | --- | --- | --- | --- | --- |
|  | Estimate | SE | p | AIC | Estimate | SE | p | AIC | Estimate | SE | p | AIC | Estimate | SE | p | AIC |
|  |  |  |  |  |  |  |  |  |  |  |  |  |  |  |  |  |
| DNA | -0.0000 | 0.0010 | 0.924 | -312.198 | -0.073 | 0.0869 | 0.098 | 389.389 | -0.0922 | 0.0636 | 0.271 | 188.298 | -0.0347 | 0.0392 | 0.378 | 153.385 |
| Intercept | 0.0045 | 0.009 | <0.0001 |  | -3.500 | 0.0834 | <0.0001 |  | 2.467 | 0.0605 | <0.0001 |  | -3.4446 | 0.0370 | <0.0001 |  |
| CD | 0.0002 | 0.0010 | 0.871 | -142.327 | 0.1998 | 0.629 | 0.002 | 396.538 | 0.0715 | 0.0457 | 0.118 | 207.936 | 0.0723 | 0.0332 | 0.034 | 149.980 |
| Intercept | -7.600 | 0.000 | <0.0001 |  | -2.764 | 00595 | <0.0001 |  | 2.6338 | 0.0424 | <0.0001 |  | -3.426 | 0.0311 | <0.0001 |  |
| WY | -0.00012 | 0.0000 | <0.0001 | -828.267 | -0.0235 | 0.0019 | <0.0001 | 358.183 | -0.0102 | 0.0007 | <0.0001 | 168.458 | -0.0092 | 0.0008 | <0.0001 | 138.648 |
| Intercept | 0.01304 | 0.00127 | <0.0001 |  | -1.794 | 0.0803 | <0.0001 |  | 3.454 | 0.0479 | <0.0001 |  | -2.844 | 0.0544 | <0.0001 |  |
|  |  |  |  |  |  |  |  |  |  |  |  |  |  |  |  |  |
|  |  |  |  | -285.182 |  |  |  | 364.815 |  |  |  | 174.069 |  |  |  | 147.742 |
| DNA | -0.00055 | 0.0008 | 0.504 |  | -0.0699 | 0.0702 | 0.340 |  | -0.0761 | 0.0707 | 0.323 |  | -0.0342 | 0.0341 | 0.320 |  |
| CD | -0.00064 | 0.0008 | 0.440 |  | 0.0371 | 0.0716 | 0.614 |  | -0.0156 | 0.0717 | 0.835 |  | 0.00121 | 0.0341 | 0.972 |  |
| WY | -0.00013 | 0.0000 | <0.0001 |  | -0.024 | 0.0019 | <0.0001 |  | -0.0125 | 0.0008 | <0.0001 |  | -0.0092 | 0.0009 | <0.0001 |  |
| Intercept | 0.01332 | 0.00135 | <0.0001 |  | -1.789 | 0.0862 | <0.0001 |  | 3.8943 | 0.0749 | <0.0001 |  | -2.8373 | 0.0556 | <0.0001 |  |
|  |  |  |  |  |  |  |  |  |  |  |  |  |  |  |  |  |
|  |  |  |  | -740.139 |  |  |  | 388.854 |  |  |  | 202.744 |  |  |  | 186.433 |
| DNA | 0.00198 | 0.00188 | 0.307 |  | -0.1291 | 0.0708 | 0.123 |  | -0.0029 | 0.0962 | 0.976 |  | -0.0003 | 0.0041 | 0.798 |  |
| CD | -0.00015 | 0.00196 | 0.940 |  | 0.1894 | 0.0788 | 0.055 |  | 0.0253 | 0.1037 | 0.813 |  | 0.00014 | -0.0002 | 0.436 |  |
| WY | -0.00014 | 0.0000 | <0.0001 |  | -0.0235 | 0.0011 | <0.0001 |  | -0.0141 | 0.0007 | <0.0001 |  | -0.00004 | -0.00005 | <0.0001 |  |
| DNA × CD | 0.00160 | 0.00193 | 0.416 |  | -0.0784 | 0.0779 | 0.354 |  | 0.0767 | 0.1011 | 0.469 |  | 0.00021 | 0.00137 | 0.806 |  |
| DNA × WY | -0.00004 | 0.00002 | 0.045 |  | -0.0028 | 0.0014 | 0.075 |  | -0.0023 | 0.0009 | 0.016 |  | 0.000000 | 0.00000 | 0.279 |  |
| DNA × CD × WY | -0.00003 | 0.00003 | 0.128 |  | 0.0022 | 0.0015 | 0.174 |  | -0.0021 | 0.0009 | 0.027 |  | 0.000000 | 0.00000 | 0.630 |  |
| Intercept | 0.01373 | 0.00172 | <0.0001 |  | -1.5909 | 0.0611 | <0.0001 |  | 3.9508 | 0.0869 | <0.0001 |  | 0.003258 | -0.00031 | <0.0001 |  |

^1^response variable was ln transformed

Table S1b Throughfall – Flux (mg m^-2^ sampling date^-1^)

|  | K flux^1^ | | | | Ca flux^1^ | | | | Mg flux^1^ | | | | S flux^1^ | | | |
| --- | --- | --- | --- | --- | --- | --- | --- | --- | --- | --- | --- | --- | --- | --- | --- | --- |
|  | Estimate | SE | p | AIC | Estimate | SE | p | AIC | Estimate | SE | p | AIC | Estimate | SE | p | AIC |
| Simple models |  |  |  |  |  |  |  |  |  |  |  |  |  |  |  |  |
| DNA | -0.1484 | 0.0651 | 0.054 | 268.920 | -0.191 | 0.0554 | 0.005 | 182.712 | -0.2637 | 0.10112 | 0.033 | 201.882 | -0.2069 | 0.07772 | 0.027 | 205.882 |
| Intercept | 5.740 | 0.6263 | <0.0001 |  | 4.992 | 0.0529 | <0.0001 |  | 5.621 | 0.0964 | <0.0001 |  | 5.54447 | 0.74065 | <0.0001 |  |
| CD | 0.022 | 0.0864 | 0.803 | 272.238 | -0.069 | 0.0969 | 0.492 | 184.260 | -0.0982 | 0.1332 | 0.483 | 206.054 | -0.1733 | 0.0687 | 0.809 | 221.806 |
| Intercept | 5.715 | 0.0783 | <0.0001 |  | 5.264 | 0.0914 | <0.0001 |  | 5.810 | 0.1258 |  |  | 5.1535 | 0.0687 | <0.0001 |  |
|  |  |  |  |  |  |  |  |  |  |  |  |  |  |  |  |  |
| Complex model |  |  |  | 272.296 |  |  |  | 181.762 |  |  |  | 202.834 |  |  |  | 208.275 |
| DNA | -0.14665 | 0.06969 | 0.077 |  | -0.2155 | 0.0658 | 0.010 |  | -0.29267 | 0.10337 | 0.025 |  | -0.22724 | 0.07901 | 0.019 |  |
| CD | 0.012551 | 0.074351 | 0.871 |  | -0.0969 | 0.0667 | 0.182 |  | -0.13960 | 0.10442 | 0.222 |  | -0.08757 | 0.07989 | 0.303 |  |
| Intercept | 5.740915 | 0.066534 | <0.0001 |  | 5.0699 | 0.0615 | <0.0001 |  | 5.62893 | 0.09651 | <0.0001 |  | 5.537514 | 0.07372 | <0.0001 |  |
|  |  |  |  |  |  |  |  |  |  |  |  |  |  |  |  |  |
| Maximal model |  |  |  | 277.974 |  |  |  | 180.807 |  |  |  | 197.673 |  |  |  | 216.719 |
| DNA | -0.17079 | 0.08177 | 0.039 |  | -0.2843 | 0.04027 | <0.0001 |  | -0.35656 | 0.05544 | 0.001 |  | -0.28003 | 0.06132 | <0.0001 |  |
| CD | 0.00836 | 0.09072 | 0.927 |  | -0.0334 | 0.0416 | 0.432 |  | -.024371 | 0.05699 | 0.686 |  | -0.01229 | 0.06307 | 0.846 |  |
| DNA × CD | -0.04964 | 0.08787 | 0.574 |  | -0.1745 | 0.0407 | <0.0001 |  | -0.25412 | 0.05571 | 0.005 |  | -0.19997 | 0.06169 | 0.002 |  |
| Intercept | 5.76542 | 0.07447 | <0.0001 |  | 4.7855 | 0.3711 | <0.0001 |  | 5.233972 | 0.05108 | <0.0001 |  | 5.356309 | 0.05646 | <0.0001 |  |

|  | Fe flux^1^ | | | | Mn flux^1^ | | | | Na flux^1^ | | | | Si flux^1^ | | | |
| --- | --- | --- | --- | --- | --- | --- | --- | --- | --- | --- | --- | --- | --- | --- | --- | --- |
|  | Estimate | SE | p | AIC | Estimate | SE | p | AIC | Estimate | SE | p | AIC | Estimate | SE | p | AIC |
| Simple models |  |  |  |  |  |  |  |  |  |  |  |  |  |  |  |  |
| DNA | -0.230 | 0.138 | 0.108 | 373.514 | -0.1319 | 0.06816 | 0.059 | 353.221 | -0.1598 | 0.07889 | 0.078 | 196.795 | 0.05216 | 0.048267 | 0.291 | 216.342 |
| Intercept | -2.511 | 0.1319 | <0.0001 |  | 0.4102 | 0.0651 | <0.0001 |  | 6.9104 | 0.7513 | <0.0001 |  | 0.54168 | 0.04546 | <0.0001 |  |
| CD | -0.0367 | 0.1742 | 0.833 | 383.251 | 0.01938 | 0.757 | 0.803 | 356.290 | -0.084 | 0.0809 | 0.326 | 186.931 | -0.1028 | 0.0496 | 0.043 | 208.916 |
| Intercept | -2.974 | 0.1644 | <0.0001 |  | 0.415 | 0.0711 | <0.0001 |  | 7.201 | 0.0757 | <0.0001 |  | 0.5986 | 0.0463 | <0.0001 |  |
|  |  |  |  |  |  |  |  |  |  |  |  |  |  |  |  |  |
| Complex model |  |  |  | 366.740 |  |  |  | 356.472 |  |  |  | 197.421 |  |  |  | 209.465 |
| DNA | -0.25081 | 0.14927 | 0.124 |  | -0.13700 | 0.72173 | 0.065 |  | -0.18557 | 0.07758 | 0.045 |  | 0.115125 | 0.080247 | 0.193 |  |
| CD | -0.02522 | 0.15187 | 0.872 |  | -0.01727 | 0.07374 | 0.816 |  | -0.12994 | 0.07894 | 0.140 |  | -0.11524 | 0.082106 | 0.201 |  |
| Intercept | -3.4560 | 0.13862 | <0.0001 |  | 0.40648 | 0.06799 | <0.0001 |  | 6.898763 | 0.07246 | <0.0001 |  | 0.770592 | 0.075123 | <0.0001 |  |
|  |  |  |  |  |  |  |  |  |  |  |  |  |  |  |  |  |
| Maximal model |  |  |  | 366.960 |  |  |  | 356.976 |  |  |  | 194.294 |  |  |  | 217.216 |
| DNA | -0.32832 | 0.15965 | 0.074 |  | -0.17058 | 0.06858 | 0.020 |  | 0.000141 | 0.00221 | <0.0001 |  | 0.052819 | 0.051115 | 0.306 |  |
| CD | 0.04739 | 0.16674 | 0.784 |  | 0.06133 | 0.72157 | 0.403 |  | -0.00001 | 0.00034 | 0.417 |  | -0.092170 | 0.052112 | 0.082 |  |
| DNA × CD | -0.21623 | 0.16262 | 0.224 |  | -0.15231 | 0.07053 | 0.040 |  | 0.000430 | 0.00045 | <0.0001 |  | -0.003022 | 0.051756 | 0.954 |  |
| Intercept | -3.55039 | 0.14685 | <0.0001 |  | 0.39506 | 0.06224 | <0.0001 |  | 0.001902 | 0.00014 | <0.0001 |  | 0.565839 | 0.047366 | <0.0001 |  |

^1^response variable was ln transformed

Table S2. Net Throughfall flux (TF flux minus BP flux) (mg m^-2^ sampling date^-1^)

|  | K flux^1^ | | | | Ca flux^1^ | | | | Mg flux^1^ | | | | S flux^1^ | | | |
| --- | --- | --- | --- | --- | --- | --- | --- | --- | --- | --- | --- | --- | --- | --- | --- | --- |
|  | Estimate | SE | p | AIC | Estimate | SE | p | AIC | Estimate | SE | p | AIC | Estimate | SE | p | AIC |
| Simple models |  |  |  |  |  |  |  |  |  |  |  |  |  |  |  |  |
| DNA | -.256645 | .071284 | .001 | 277.840 | -.195534 | .069742 | .021 | 225.011 | -.263233 | .113436 | .046 | 243.951 | -.233345 | .083993 | .007 | 224.957 |
| Intercept | 6.347001 | .067694 | .000 |  | 5.001765 | .066580 | .000 |  | 5.594938 | .108175 | .000 |  | 5.241829 | .078916 | .000 |  |
| CD | 5.730660 | .068292 | .664 | 283.119 | -.063406 | .098975 | .538 | 216.086 | -.092683 | .143174 | .534 | 247.225 | -.103133 | .091603 | .000 | 207.542 |
| Intercept | .033033 | .075489 | .000 |  | 5.195046 | .093320 | .000 |  | 5.772132 | .135092 | .000 |  | 5.894341 | .087463 | .000 |  |
|  |  |  |  |  |  |  |  |  |  |  |  |  |  |  |  |  |
| Complex model |  |  |  | 281.261 |  |  |  | 215.539 |  |  |  | 245.022 |  |  |  | 217.049 |
| DNA | -.161031 | .072569 | .065 |  | -.203179 | .065898 | .011 |  | -.291224 | .114948 | .034 |  | -.270260 | .087896 | .016 |  |
| CD | .014561 | .077398 | .856 |  | -.097455 | .066761 | .173 |  | -.139245 | .116209 | .265 |  | -.154091 | .087353 | .115 |  |
| Intercept | 5.717197 | .069251 | .000 |  | 5.022711 | .061684 | .000 |  | 5.592227 | .107453 | .000 |  | 5.406061 | .080096 | .000 |  |
|  |  |  |  |  |  |  |  |  |  |  |  |  |  |  |  |  |
| Maximal model |  |  |  | 293.842 |  |  |  | 212.813 |  |  |  | 241.338 |  |  |  | 213.139 |
| DNA | -.277208 | .083230 | .002 |  | -.292150 | .047606 | .001 |  | -.401862 | .067272 | .000 |  | -.343484 | .056726 | .000 |  |
| CD | .054557 | .088746 | .542 |  | -.034213 | .049397 | .513 |  | -.027698 | .069295 | .700 |  | -.062643 | .058476 | .291 |  |
| DNA × CD | -.115146 | .086378 | .189 |  | -.179799 | .048212 | .009 |  | -.286738 | .067779 | .003 |  | -.215615 | .057306 | .001 |  |
| Intercept | 5.832700 | .076323 | .000 |  | 4.761031 | .043820 | .000 |  | 5.199141 | .061766 | .000 |  | 5.082799 | .052227 | .000 |  |

|  | Fe flux^1^ | | | | Mn flux^1^ | | | | Na flux^1^ | | | | Si flux^1^ | | | |
| --- | --- | --- | --- | --- | --- | --- | --- | --- | --- | --- | --- | --- | --- | --- | --- | --- |
|  | Estimate | SE | p | AIC | Estimate | SE | p | AIC | Estimate | SE | p | AIC | Estimate | SE | p | AIC |
| Simple models |  |  |  |  |  |  |  |  |  |  |  |  |  |  |  |  |
| DNA | -.218819 | .142167 | .151 | 394.294 | -.166669 | .076888 | .509 | 391.152 | -5.7358E-5 | .082728 | .999 | 251.548 | -.086737 | .064149 | .185 | 307.100 |
| Intercept | -1.783835 | .130482 | .000 |  | .384575 | .073603 | .364 |  | 7.041980 | .078828 | .000 |  | .507919 | .060987 | .000 |  |
| CD | .235801 | .117851 | .077 | 390.688 | .004454 | .083643 | .958 | 387.088 | -.085358 | .078845 | .304 | 250.504 | -.112764 | .076040 | .176 | 306.245 |
| Intercept | -1.697442 | .112151 | .000 |  | .272308 | .078294 | .004 |  | 7.001858 | .073948 | .000 |  | .590490 | .071801 | .000 |  |
|  |  |  |  |  |  |  |  |  |  |  |  |  |  |  |  |  |
| Complex model |  |  |  | 389.817 |  |  |  | 386.506 |  |  |  | 253.640 |  |  |  | 307.707 |
| DNA | -.226415 | .115034 | .088 |  | -.158172 | .075512 | .060 |  | -.021672 | .081796 | .798 |  | -.110462 | .064715 | .096 |  |
| CD | .188037 | .110789 | .132 |  | -.031058 | .077681 | .697 |  | -.088429 | .083148 | .319 |  | -.122242 | .065966 | .072 |  |
| Intercept | -1.749341 | .105842 | .000 |  | .268033 | .072175 | .003 |  | 7.012967 | .076545 | .000 |  | .494073 | .060050 | .000 |  |
|  |  |  |  |  |  |  |  |  |  |  |  |  |  |  |  |  |
| Maximal model |  |  |  | 392.186 |  |  |  | 388.104 |  |  |  | 256.541 |  |  |  | 310.886 |
| DNA | -.253629 | .105949 | .046 |  | -.202664 | .068374 | .018 |  | .005495 | .095181 | .956 |  | -.106833 | .082552 | .260 |  |
| CD | .191306 | .102432 | .099 |  | .025105 | .074466 | .744 |  | -.092836 | .099105 | .391 |  | -.140608 | .083331 | .148 |  |
| DNA × CD | -.082151 | .102918 | .448 |  | -.134673 | .072950 | .097 |  | .031750 | .097185 | .757 |  | .005932 | .082144 | .945 |  |
| Intercept | -1.772897 | .094920 | .000 |  | .234313 | .060507 | .006 |  | 7.075030 | .086436 | .000 |  | .608734 | .074598 | .000 |  |

^1^response variable was ln transformed

Table S3a. Stemflow - Concentration (mg l^-1^)

|  | K concentration^1^ | | | | Ca concentration^1^ | | | | Mg concentration^1^ | | | | S concentration^1^ | | | |
| --- | --- | --- | --- | --- | --- | --- | --- | --- | --- | --- | --- | --- | --- | --- | --- | --- |
|  | Estimate | SE | p | AIC | Estimate | SE | p | AIC | Estimate | SE | p | AIC | Estimate | SE | p | AIC |
| Simple models |  |  |  |  |  |  |  |  |  |  |  |  |  |  |  |  |
| DNA | .143249 | .166739 | .414 | 208.594 | .140709 | .088807 | .127 | 180.595 | .009808 | .122741 | .936 | 261.232 | -.092295 | .082522 | .287 | 224.820 |
| Intercept | 3.035281 | .156720 | .000 |  | 2.084573 | .085362 | .000 |  | 1.265198 | .118890 | .000 |  | 2.229766 | .076324 | .000 |  |
| CD | .096412 | .175400 | .597 | 208.932 | .079489 | .116168 | .574 | 200.172 | -.108770 | .174443 | .558 | 266.513 | .028657 | .094123 | .767 | 223.633 |
| Intercept | 3.046714 | .165584 | .000 |  | 2.048989 | .114731 | .003 |  | 1.125867 | .164817 | .001 |  | 2.260531 | .087086 | .000 |  |
| WY | -3.626E-5 | 1.4009E-5 | .014 | 224.971 | -5.045E-5 | 1.1734E-5 | .000 | 211.551 | -5.9418E-5 | 2.0326E-5 | .004 | 273.273 | -8.6925E-6 | 1.3752E-5 | .533 | 241.089 |
| Intercept | 3.216789 | .160696 | .000 |  | 2.101182 | .076007 | .000 |  | 1.475109 | .129945 | .000 |  | 2.266716 | .098077 | .000 |  |
|  |  |  |  |  |  |  |  |  |  |  |  |  |  |  |  |  |
| Complex model |  |  |  | 230.150 |  |  |  | 221.478 |  |  |  | 418.354 |  |  |  | 246.589 |
| DNA | .120107 | .161145 | .474 |  | .136706 | .096370 | .158 |  | -.227202 | .304001 | .470 |  | -.102809 | .095393 | .313 |  |
| CD | .108949 | .156619 | .504 |  | .069392 | .093718 | .460 |  | -.059889 | .292856 | .841 |  | .015438 | .093201 | .873 |  |
| WY | -3.206E-5 | 1.4411E-5 | .028 |  | -3.48E-5 | 1.493E-5 | .021 |  | -3.7230E-5 | 4.7599E-5 | .438 |  | -1.0456E-5 | 1.3997E-5 | .461 |  |
| Intercept | 3.208195 | .161869 | .000 |  | 2.040558 | .099936 | .000 |  | .823985 | .374991 | .041 |  | 2.303527 | .100846 | .000 |  |
|  |  |  |  |  |  |  |  |  |  |  |  |  |  |  |  |  |
| Maximal model |  |  |  | 262.247 |  |  |  | 257.815 |  |  |  | 290.515 |  |  |  | 291.477 |
| DNA | .224356 | .167668 | .183 |  | .172141 | .103634 | .111 |  | -.084669 | .134964 | .531 |  | -.214990 | .102941 | .068 |  |
| CD | -.010353 | .143975 | .943 |  | .143449 | .094804 | .145 |  | -.024779 | .132207 | .852 |  | -.032421 | .098098 | .750 |  |
| WY | -9.422E-5 | 2.466E-5 | .000 |  | -6.37E-5 | 3.224E-5 | .054 |  | -4.1772E-5 | 3.5382E-5 | .240 |  | -9.8084E-5 | 2.5375E-5 | .000 |  |
| DNA × CD | .339973 | .167640 | .045 |  | .079594 | .096570 | .419 |  | -.093066 | .135535 | .493 |  | -.139580 | .102961 | .207 |  |
| DNA × WY | -6.648E-5 | 2.7262E-5 | .017 |  | -5.99E-5 | 3.76E-5 | .118 |  | -4.4202E-5 | 3.7959E-5 | .247 |  | -5.8542E-5 | 2.7177E-5 | .035 |  |
| DNA × CD × WY | -6.426E-5 | 2.3987E-5 | .009 |  | -7.79E-5 | 2.76E-5 | .007 |  | -4.9841E-5 | 3.5022E-5 | .158 |  | -4.8408E-5 | 2.46848E-5 | .054 |  |
| Intercept | 3.273612 | .152110 | .000 |  | 2.18645 | .095129 | .000 |  | 2.330528 | .140452 | .000 |  | 2.378971 | .095215 | .000 |  |

^1^response variable was ln transformed

Table S3a. Stemflow - Concentration (mg l^-1^)

|  | Fe concentration^1^ | | | | Mn concentration^1^ | | | | Na concentration^1^ | | | | Si concentration^1^ | | | |
| --- | --- | --- | --- | --- | --- | --- | --- | --- | --- | --- | --- | --- | --- | --- | --- | --- |
|  | Estimate | SE | p | AIC | Estimate | SE | p | AIC | Estimate | SE | p | AIC | Estimate | SE | p | AIC |
|  |  |  |  |  |  |  |  |  |  |  |  |  |  |  |  |  |
| DNA | .128311 | .094488 | .176 | 281.007 | -.030818 | .243075 | .901 | 294.500 | -.113011 | .065452 | .150 | 204.425 | .130687 | .065821 | .069 | 176.592 |
| Intercept | -3.142543 | .087471 | .000 |  | -2.495891 | .235276 | .000 |  | 4.061891 | .062552 | .000 |  | -2.030833 | .063664 | .000 |  |
| CD | -.163005 | .080522 | .064 | 286.964 | -.277766 | .230171 | .263 | 293.165 | .017388 | .066479 | .797 | 210.113 | -.085703 | .058470 | .151 | 176.256 |
| Intercept | -3.172686 | .076774 | .000 |  | -2.479341 | .219519 | .000 |  | 3.858346 | .061706 | .000 |  | -2.180087 | .054246 | .000 |  |
| WY | -3.9907E-5 | 1.3141E-5 | .006 | 293.470 | -3.013E-5 | 2.0626E-5 | .154 | 280.115 | -5.610E-5 | 1.7752E-5 | .002 | 259.028 | -5.33442E-5 | 1.18142E-5 | .000 | 194.708 |
| Intercept | -2.985058 | .083186 | .000 |  | -1.279428 | .153913 | .000 |  | 3.471111 | .117379 | .000 |  | -2.499977 | .090689 | .000 |  |
|  |  |  |  |  |  |  |  |  |  |  |  |  |  |  |  |  |
|  |  |  |  | 296.683 |  |  |  | 281.469 |  |  |  | 253.428 |  |  |  | 188.814 |
| DNA | -.017935 | .070824 | .804 |  | -.145560 | .154293 | .390 |  | -.012438 | .116578 | .915 |  | .106261 | .055854 | .083 |  |
| CD | -.137888 | .069943 | .067 |  | -.222802 | .154626 | .211 |  | .020313 | .117587 | .863 |  | -.094900 | .057595 | .129 |  |
| WY | -4.0056E-5 | 1.2936E-5 | .006 |  | -3.248E-5 | 2.060E-5 | .128 |  | -6.284E-5 | 1.7784E-5 | .001 |  | -2.22084E-5 | 9.6006E-6 | .026 |  |
| Intercept | -3.008446 | .081612 | .000 |  | -1.244771 | .153796 | .000 |  | 3.553497 | .130128 | .000 |  | -2.190619 | .061570 | .000 |  |
|  |  |  |  |  |  |  |  |  |  |  |  |  |  |  |  |  |
|  |  |  |  | 333.900 |  |  |  | 313.610 |  |  |  | 259.220 |  |  |  | 235.371 |
| DNA | -.009231 | .097263 | .925 |  | -.180191 | .135829 | .246 |  | -.137283 | .108380 | .237 |  | .109909 | .104364 | .294 |  |
| CD | -.125143 | .084157 | .156 |  | -.170059 | .128378 | .262 |  | .056238 | .106136 | .611 |  | -.128351 | .089598 | .153 |  |
| WY | -9.2375E-5 | 2.8245E-5 | .003 |  | -.000134 | 4.0182E-5 | .002 |  | -5.197E-5 | 2.631E-5 | .055 |  | -8.9786E-5 | 1.8583E-5 | .000 |  |
| DNA × CD | -.047944 | .100405 | .638 |  | -.195078 | .135932 | .208 |  | -.134381 | .109908 | .250 |  | .135079 | .106472 | .206 |  |
| DNA × WY | -5.9806E-5 | 3.1607E-5 | .068 |  | -7.709E-5 | 4.5224E-5 | .097 |  | -4.426E-5 | 2.7803E-5 | .118 |  | -3.2561E-5 | 2.1167E-5 | .128 |  |
| DNA × CD × WY | -5.9697E-5 | 2.8341E-5 | .043 |  | -9.011E-5 | 4.1014E-5 | .035 |  | -4.015E-5 | 2.5099E-5 | .117 |  | -5.6192E-5 | 1.9447E-5 | .005 |  |
| Intercept | -2.957066 | .089579 | .000 |  | -1.206752 | .126847 | .000 |  | 4.225522 | .101449 | .000 |  | -2.411395 | .102589 | .000 |  |

^1^response variable was ln transformed

Table S3b. Stemflow - Flux (mg m^-2^ sampling day^-1^)

|  | K flux^1^ | | | | Ca flux^1^ | | | | Mg flux^1^ | | | | S flux^1^ | | | |
| --- | --- | --- | --- | --- | --- | --- | --- | --- | --- | --- | --- | --- | --- | --- | --- | --- |
|  | Estimate | SE | p | AIC | Estimate | SE | p | AIC | Estimate | SE | p | AIC | Estimate | SE | p | AIC |
| Simple models |  |  |  |  |  |  |  |  |  |  |  |  |  |  |  |  |
| DNA | .419426 | .251406 | .153 | 302.784 | .188091 | .301681 | .559 | 308.822 | .047623 | .365892 | .901 | 369.298 | .130319 | .312414 | .694 | 351.973 |
| Intercept | .180196 | .233028 | .472 |  | -1.663998 | .279242 | .000 |  | -1.354822 | .338393 | .010 |  | -1.230756 | .289260 | .008 |  |
| CD | .716608 | .156075 | .004 | 296.338 | .642503 | .189732 | .016 | 303.864 | .722065 | .244258 | .034 | 364.128 | .861571 | .194447 | .004 | 342.103 |
| Intercept | -.142034 | .126514 | .307 |  | -1.861680 | .154799 | .000 |  | -1.522802 | .200516 | .001 |  | -1.175459 | .156591 | .000 |  |
|  |  |  |  |  |  |  |  |  |  |  |  |  |  |  |  |  |
| Complex model |  |  |  | 296.839 |  |  |  | 304.546 |  |  |  | 364.353 |  |  |  | 342.711 |
| DNA | .183425 | .149549 | .275 |  | -.046108 | .217297 | .841 |  | -.221259 | .240485 | .412 |  | -.184723 | .183660 | .362 |  |
| CD | .632683 | .166758 | .013 |  | .656017 | .239741 | .047 |  | .797918 | .263899 | .042 |  | .937941 | .206782 | .007 |  |
| Intercept | -.071644 | .137742 | .625 |  | -1.908103 | .203698 | .000 |  | -1.639675 | .227216 | .002 |  | -1.253394 | .166641 | .001 |  |
|  |  |  |  |  |  |  |  |  |  |  |  |  |  |  |  |  |
| Maximal model |  |  |  | 295.185 |  |  |  | 302.583 |  |  |  | 361.947 |  |  |  | 339.666 |
| DNA | .443712 | .352319 | .226 |  | .230085 | .700162 | .762 |  | .238165 | .766353 | .776 |  | .460123 | .509638 | .410 |  |
| CD | .430272 | .395081 | .292 |  | .354544 | .787216 | .680 |  | .285653 | .861925 | .762 |  | .227691 | .572230 | .708 |  |
| DNA × CD | -.554518 | .526635 | .309 |  | -.426527 | 1.036920 | .705 |  | -.714409 | 1.132670 | .573 |  | -1.030383 | .761881 | .238 |  |
| Intercept | .434983 | .304850 | .174 |  | -1.670253 | .599376 | .059 |  | -1.253602 | .654660 | .151 |  | -.681924 | .440640 | .186 |  |

|  | Fe flux^1^ | | | | Mn flux^1^ | | | | Na flux^1^ | | | | Si flux^1^ | | | |
| --- | --- | --- | --- | --- | --- | --- | --- | --- | --- | --- | --- | --- | --- | --- | --- | --- |
|  | Estimate | SE | p | AIC | Estimate | SE | p | AIC | Estimate | SE | p | AIC | Estimate | SE | p | AIC |
| Simple models |  |  |  |  |  |  |  |  |  |  |  |  |  |  |  |  |
| DNA | .307197 | .290939 | .335 | 364.939 | .203073 | .256182 | .461 | 370.624 | .061996 | .325935 | .857 | 345.245 | .237364 | .287041 | .443 | 288.887 |
| Intercept | -6.410307 | .270063 | .000 |  | -4.799339 | .237672 | .000 |  | .305762 | .301589 | .356 |  | -5.160502 | .266424 | .000 |  |
| CD | .701369 | .208352 | .022 | 358.532 | .749717 | .122004 | .000 | 360.583 | .632187 | .230705 | .039 | 340.429 | .643854 | .203358 | .019 | 283.268 |
| Intercept | -6.654130 | .168067 | .000 |  | -5.135469 | .099556 | .000 |  | .128661 | .188930 | .525 |  | -5.438640 | .164145 | .000 |  |
|  |  |  |  |  |  |  |  |  |  |  |  |  |  |  |  |  |
| Complex model |  |  |  | 360.624 |  |  |  | 356.724 |  |  |  | 343.741 |  |  |  | 284.573 |
| DNA | .014462 | .184169 | .940 |  | -.109271 | .116763 | .367 |  | -.143273 | .219409 | .545 |  | -.024800 | .215546 | .913 |  |
| CD | .741524 | .206958 | .016 |  | .799720 | .129003 | .000 |  | .717095 | .244373 | .036 |  | .658399 | .241958 | .044 |  |
| Intercept | -6.768277 | .167656 | .000 |  | -5.164090 | .109226 | .000 |  | .230850 | .202693 | .311 |  | -5.432906 | .196577 | .000 |  |
|  |  |  |  |  |  |  |  |  |  |  |  |  |  |  |  |  |
| Maximal model |  |  |  | 354.749 |  |  |  | 377.854 |  |  |  | 333.310 |  |  |  | 280.618 |
| DNA | 1.031792 | .536083 | .135 |  | .071676 | .545725 | .914 |  | .271729 | .832386 | .765 |  | .816113 | .536965 | .199 |  |
| CD | -.459498 | .601998 | .493 |  | .398692 | .613757 | .616 |  | .207578 | .936216 | .838 |  | -.282416 | .602965 | .663 |  |
| DNA × CD | -1.663070 | .800596 | .115 |  | -.497504 | .806778 | .632 |  | -.765365 | 1.230096 | .577 |  | -1.351334 | .802188 | .163 |  |
| Intercept | -5.716250 | .462997 | .000 |  | -5.396679 | .466306 | .034 |  | .416975 | .710966 | .598 |  | -4.691623 | .463931 | .000 |  |

^1^response variable was ln transformed

Table S4a. Forest floor leachate - Concentration (mg l^-1^)

|  | K concentration^1^ | | | | Ca concentration^1^ | | | | Mg concentration^1^ | | | | S concentration^1^ | | | |
| --- | --- | --- | --- | --- | --- | --- | --- | --- | --- | --- | --- | --- | --- | --- | --- | --- |
|  | Estimate | SE | p | AIC | Estimate | SE | p | AIC | Estimate | SE | p | AIC | Estimate | SE | p | AIC |
| Simple models |  |  |  |  |  |  |  |  |  |  |  |  |  |  |  |  |
| DNA | -.095498 | .083579 | .296 | 180.733 | -.066583 | .085370 | .460 | 142.095 | -.124065 | .066202 | .088 | 148.768 | -.095234 | .062498 | .162 | 155.725 |
| Intercept | 3.066697 | .079357 | .000 |  | 2.121856 | .081334 | .000 |  | 2.493701 | .062987 | .000 |  | 2.201067 | .059412 | .000 |  |
| CD | .102236 | .055163 | .075 | 185.891 | -.018146 | .088671 | .843 | 142.623 | .075861 | .069251 | .294 | 153.241 | .059208 | .066385 | .393 | 164.512 |
| Intercept | 3.279451 | .052399 | .000 |  | 2.114951 | .083934 | .000 |  | 2.500774 | .065712 | .000 |  | 2.239231 | .063022 | .000 |  |
| Organic layer | .104760 | .081900 | .246 | 180.568 | .039085 | .091776 | .683 | 142.410 | .162028 | .081434 | .104 | 187.646 | .121561 | .059074 | .067 | 153.689 |
| Intercept | 3.053722 | .076430 | .000 |  | 2.117620 | .083574 | .000 |  | 1.958068 | .074999 | .000 |  | 2.198198 | .055268 | .000 |  |
| WY | .003062 | .001214 | .015 | 188.727 | -.008404 | .001105 | .000 | 116.918 | -.003339 | .001291 | .011 | 166.244 | -.003975 | .001130 | .001 | 168.016 |
| Intercept | 2.930955 | .096839 | .000 |  | 3.183450 | .079829 | .000 |  | 2.800024 | .105754 | .000 |  | 2.487652 | .098144 | .000 |  |
|  |  |  |  |  |  |  |  |  |  |  |  |  |  |  |  |  |
| Complex model |  |  |  | 192.870 |  |  |  | 127.136 |  |  |  |  |  |  |  | 180.675 |
| DNA | -.044048 | .103433 | .687 |  | -.019861 | .091025 | .834 |  | -.030295 | .071748 | .682 | 166.801 | -.019976 | .059894 | .745 |  |
| CD | .099957 | .083240 | .281 |  | -.007798 | .073292 | .918 |  | .044029 | .057633 | .463 |  | .056185 | .048039 | .267 |  |
| WY | .076297 | .106033 | .500 |  | .012219 | .092805 | .899 |  | .127590 | .071539 | .105 |  | .038157 | .061661 | .551 |  |
| Organic layer | .002906 | .001281 | .029 |  | -.007379 | .001104 | .000 |  | -.005594 | .001201 | .000 |  | -.007258 | .001554 | .000 |  |
| Intercept | 2.920517 | .095184 | .000 |  | 3.179512 | .084835 | .000 |  | 2.787559 | .089330 | .000 |  | 2.706975 | .110676 | .000 |  |
|  |  |  |  |  |  |  |  |  |  |  |  |  |  |  |  |  |
| Maximal model |  |  |  | 239.329 |  |  |  | 189.046 |  |  |  | 231.723 |  |  |  | 231.041 |
| DNA | -.685077 | .767543 | .386 |  | -.322630 | .499223 | .524 |  | .143101 | .758847 | .852 |  | -.223305 | .834983 | .797 |  |
| CD | .953085 | .968521 | .343 |  | -.041150 | .611389 | .947 |  | -.076736 | .807447 | .925 |  | .331771 | 1.056039 | .764 |  |
| Organic layer | -1.04613 | 1.017014 | .321 |  | -.267589 | .653340 | .686 |  | -.012433 | .009724 | .205 |  | -.346018 | 1.101375 | .763 |  |
| WY | .001913 | .013629 | .890 |  | -.011996 | .009004 | .191 |  | -.019581 | .800609 | .981 |  | -.006662 | .011405 | .568 |  |
| DNA × CD | .705982 | 1.019919 | .501 |  | .210605 | .647928 | .748 |  | -.377479 | .922779 | .686 |  | .129365 | 1.116764 | .912 |  |
| DNA × OL | -1.47836 | 1.161320 | .223 |  | -.635178 | .747554 | .405 |  | -.012654 | .010335 | .224 |  | -.620925 | 1.267998 | .641 |  |
| DNA × WY | -.004748 | .014331 | .744 |  | -.005751 | .009620 | .553 |  | .143101 | .758847 | .852 |  | -.004642 | .012041 | .705 |  |
| DNA × CD × OL ×WY | -.008368 | .028152 | .770 |  | .007051 | .018435 | .704 |  | .019537 | .019809 | .327 |  | .000187 | .023471 | .994 |  |
| Intercept | 2.288698 | .756334 | .009 |  | 2.776456 | .482940 | .000 |  | 2.396661 | .597189 | .000 |  | 2.194088 | .821132 | .035 |  |

^1^response variable was ln transformed

Table S4a. Forest floor leachate - Concentration (mg l^-1^)

|  | Fe concentration^1^ | | | | Mn concentration^1^ | | | | Na concentration^1^ | | | | Si concentration^1^ | | | |
| --- | --- | --- | --- | --- | --- | --- | --- | --- | --- | --- | --- | --- | --- | --- | --- | --- |
|  | Estimate | SE | p | AIC | Estimate | SE | p | AIC | Estimate | SE | p | AIC | Estimate | SE | p | AIC |
| Simple models |  |  |  |  |  |  |  |  |  |  |  |  |  |  |  |  |
| DNA | .021790 | .140998 | .000 | 197.463 | -.034589 | .019281 | .125 | -246.384 | -.045989 | .065189 | .500 | 119.773 | 1.764169 | .096695 | .000 | -295.922 |
| Intercept | -1.205794 | .134085 | .881 |  | .012025 | .018396 | .539 |  | 3.167491 | .062310 | .000 |  | -2.570359 | .103817 | .000 |  |
| CD | -.270892 | .120376 | .054 | 194.504 | -.026615 | .345086 | .940 | 269.579 | .078418 | .039500 | .061 | 129.037 | .119428 | .085117 | .203 | 142.307 |
| Intercept | -1.234297 | .113867 | 000 |  | -3.101325 | .320751 | .000 |  | 3.551859 | .037431 | .000 |  | -1.805395 | .080772 | .000 |  |
| Organic layer | .265464 | .117725 | .025 | 198.048 | .752171 | .229413 | .016 | 310.176 | .080828 | .062831 | .234 | 118.847 | .161905 | .040063 | .000 | 139.577 |
| Intercept | -1.283537 | .109055 | .000 |  | -2.946903 | .216304 | .000 |  | 3.204101 | .055158 | .000 |  | -1.782741 | .036038 | .000 |  |
| WY | -.002897 | .001913 | .144 | 205.396 | -.014111 | .003036 | .000 | 317.695 | -.006301 | .001036 | .000 | 102.200 | -.005701 | .001715 | .002 | 145.210 |
| Intercept | -1.196148 | .147410 | .000 |  | -2.272337 | .369169 | .000 |  | 3.804618 | .064949 | .000 |  | -1.494859 | .121971 | .000 |  |
|  |  |  |  |  |  |  |  |  |  |  |  |  |  |  |  |  |
| Complex model |  |  |  | 208.250 |  |  |  | 309.235 |  |  |  | 109.755 |  |  |  | 141.948 |
| DNA | .177295 | .176659 | .447 |  | -.103654 | .309940 | .755 |  | .070522 | .063139 | .294 |  | -.087047 | .046188 | .070 |  |
| CD | -.131826 | .141631 | .475 |  | -.221789 | .248789 | .422 |  | .075965 | .050731 | .170 |  | .039207 | .036972 | .298 |  |
| WY | .429822 | .179357 | .229 |  | .878966 | .312066 | .043 |  | .088371 | .064614 | .204 |  | .078171 | .049075 | .121 |  |
| Organic layer | -.000502 | .001977 | .831 |  | -.015311 | .003033 | .000 |  | -.006196 | .001053 | .000 |  | -.007073 | .001622 | .000 |  |
| Intercept | 3.179512 | .084835 | .229 |  | -2.235961 | .276236 | .000 |  | 3.797494 | .063562 | .000 |  | -1.415871 | .088177 | .000 |  |
|  |  |  |  |  |  |  |  |  |  |  |  |  |  |  |  |  |
| Maximal model |  |  |  | 253.759 |  |  |  | 345.422 |  |  |  | 175.426 |  |  |  | 195.461 |
| DNA | -1.927274 | .982647 | .125 |  | 1.381074 | 1.827817 | .473 |  | .407292 | .497782 | .425 |  | -.954690 | .689238 | .176 |  |
| CD | 1.825614 | 1.214896 | .213 |  | -1.055526 | 2.251719 | .653 |  | -.292870 | .613619 | .640 |  | 1.391560 | .853929 | .113 |  |
| Organic layer | -2.370668 | 1.296533 | .147 |  | 1.901079 | 2.472167 | .466 |  | .598928 | .659992 | .377 |  | -1.257327 | .947545 | .195 |  |
| WY | .012997 | .015096 | .393 |  | -.055683 | .027043 | .057 |  | -.015429 | .007862 | .055 |  | .002603 | .014023 | .854 |  |
| DNA × CD | 1.837051 | 1.292823 | .238 |  | -1.981348 | 2.385196 | .434 |  | -.248982 | .654125 | .709 |  | 1.925915 | .886715 | .037 |  |
| DNA × OL | -2.911991 | 1.478254 | .126 |  | 2.125350 | 2.728175 | .461 |  | .270960 | .747934 | .722 |  | -1.540080 | 1.024383 | .143 |  |
| DNA × WY | .007289 | .015930 | .649 |  | -.053789 | .028250 | .075 |  | -.011066 | .008345 | .191 |  | .007760 | .014521 | .597 |  |
| DNA × CD × OL ×WY | -.000279 | .030899 | .993 |  | .063432 | .054587 | .262 |  | .007278 | .016117 | .654 |  | -.034961 | .028096 | .222 |  |
| Intercept | -2.868958 | .956992 | .044 |  | -1.363393 | 1.792048 | .470 |  | 4.051459 | .484517 | .000 |  | -2.322646 | .683775 | .002 |  |

^1^response variable was ln transformed

Table S4b. Forest floor leachate – Flux (mg m^-2^ sampling date^-1^)

|  | K flux^1^ | | | | Ca flux^1^ | | | | Mg flux^1^ | | | | S flux^1^ | | | |
| --- | --- | --- | --- | --- | --- | --- | --- | --- | --- | --- | --- | --- | --- | --- | --- | --- |
|  | Estimate | SE | p | AIC | Estimate | SE | p | AIC | Estimate | SE | p | AIC | Estimate | SE | p | AIC |
| Simple models |  |  |  |  |  |  |  |  |  |  |  |  |  |  |  |  |
| DNA | .071884 | .079718 | .399 | 243.969 | .044995 | .038213 | .239 | 165.950 | -.035518 | .055468 | .534 | 187.398 | -.038990 | .055503 | .500 | 217.111 |
| Intercept | 7.752606 | .076179 | .000 |  | 6.755462 | .036475 | .000 |  | 6.557262 | .052987 | .000 |  | 6.514524 | .052986 | .000 |  |
| CD | -.012266 | .078891 | .881 | 244.678 | -.027697 | .048480 | .585 | 143.873 | .043374 | .060188 | .487 | 182.621 | .031520 | .055483 | 0.583 | 192.650 |
| Intercept | 7.730926 | .074483 | .000 |  | 6.788881 | .045785 | .000 |  | 6.706647 | .056820 | .000 |  | 6.442916 | .052382 | .000 |  |
| Organic layer | -.150590 | .083171 | .115 | 241.531 | -.032044 | .039686 | .447 | 174.947 | .022751 | .066461 | .741 | 180.997 | -.011709 | .055958 | .838 | 201.894 |
| Intercept | 7.758169 | .073419 | .000 |  | 6.493752 | .037025 | .000 |  | 6.686537 | .058703 | .000 |  | 6.463778 | .049063 | .000 |  |
|  |  |  |  |  |  |  |  |  |  |  |  |  |  |  |  |  |
| Complex model |  |  |  | 247.098 |  |  |  | 206.152 |  |  |  | 204.671 |  |  |  | 199.178 |
| DNA | -.027377 | .116057 | .823 |  | .030210 | .063862 | .649 |  | -.033848 | .078229 | .666 |  | -.039356 | .080534 | .641 |  |
| CD | .019655 | .093297 | .841 |  | -.014442 | .051336 | .786 |  | .037886 | .062885 | .548 |  | .034529 | .064724 | .611 |  |
| Organic layer | -.197412 | .127801 | .184 |  | -.105719 | .069332 | .170 |  | -.015895 | .083488 | .849 |  | -.058900 | .088552 | .529 |  |
| Intercept | 7.866624 | .085559 | .000 |  | 6.830933 | .047063 | .000 |  | 6.655234 | .057620 | .000 |  | 6.504824 | .059355 | .000 |  |
|  |  |  |  |  |  |  |  |  |  |  |  |  |  |  |  |  |
| Maximal model |  |  |  | 239.914 |  |  |  | 151.862 |  |  |  | 187.061 |  |  |  | 199.765 |
| DNA | -.435388 | .251779 | .256 |  | -.504036 | .213627 | .103 |  | -.818389 | .567945 | .273 |  | -.351126 | .418004 | .403 |  |
| CD | .039704 | .310974 | .912 |  | .230949 | .263688 | .447 |  | .754183 | .697997 | .382 |  | .146857 | .514015 | .776 |  |
| Organic layer | -.641060 | .333479 | .221 |  | -.673212 | .282710 | .099 |  | -1.085248 | .747809 | .270 |  | -.470488 | .550739 | .395 |  |
| DNA × CD | -.311845 | .337995 | .472 |  | .207562 | .286671 | .523 |  | .639740 | .759911 | .480 |  | -.090408 | .559508 | .872 |  |
| DNA × OL | -.435265 | .379777 | .393 |  | -.677314 | .322135 | .129 |  | -1.161831 | .854612 | .294 |  | -.354205 | .629168 | .575 |  |
| DNA × CD × OL | -.449076 | .508477 | .485 |  | .318995 | .430699 | .512 |  | 1.005288 | 1.133340 | .459 |  | -.095001 | .835229 | .910 |  |
| Intercept | 7.090120 | .244860 | .003 |  | 6.259132 | .207646 | .000 |  | 6.216248 | .550376 | .005 |  | 6.296497 | .405230 | .000 |  |

^1^response variable was ln transformed

Table S4b. Forest floor leachate – Flux (mg m^-2^ sampling date^-1^)

|  | Fe flux^1^ | | | | Mn flux^1^ | | | | Na flux^1^ | | | | Si flux^1^ | | | |
| --- | --- | --- | --- | --- | --- | --- | --- | --- | --- | --- | --- | --- | --- | --- | --- | --- |
|  | Estimate | SE | p | AIC | Estimate | SE | p | AIC | Estimate | SE | p | AIC | Estimate | SE | p | AIC |
| Simple models |  |  |  |  |  |  |  |  |  |  |  |  |  |  |  |  |
| DNA | .054659 | .044787 | .249 | 268.611 | -.528321 | .269420 | .089 | 468.984 | -.054831 | .056854 | .366 | 164.730 | -.074223 | .047986 | .162 | 174.801 |
| Intercept | 2.868224 | .042518 | .000 |  | 1.017043 | .255778 | .005 |  | -.054831 | .056854 | .000 |  | 2.508054 | .045751 | .000 |  |
| CD | -.025074 | .024739 | .343 | 235.328 | -.033132 | .330627 | .923 | 472.067 | .043358 | .068207 | .548 | 170.745 | .123202 | .052835 | .035 | 189.044 |
| Intercept | 2.857731 | .023392 | .000 |  | 1.052097 | .313468 | .011 |  | 7.495129 | .064369 | .000 |  | 2.632049 | .050099 | .000 |  |
| Organic layer | -.022701 | .017167 | .198 | 241.638 | .808961 | .194026 | .002 | 465.673 | -.008082 | .068392 | .909 | 165.287 | .067393 | .052270 | .235 | 175.202 |
| Intercept | 2.792446 | .016289 | .000 |  | .821293 | .177228 | .002 |  | 7.421310 | .060202 | .000 |  | 2.511896 | .047166 | .000 |  |
|  |  |  |  |  |  |  |  |  |  |  |  |  |  |  |  |  |
| Complex model |  |  |  | 285.733 |  |  |  | 461.557 |  |  |  | 164.086 |  |  |  | 186.921 |
| DNA | .036895 | .074213 | .619 |  | -.429910 | .197832 | .062 |  | -.037894 | .060063 | .542 |  | -.610113 | .375300 | .105 |  |
| CD | -.003293 | .059613 | .956 |  | -.407413 | .159067 | .034 |  | .045263 | .048271 | .370 |  | .395894 | .302178 | .191 |  |
| Organic layer | .013796 | .074913 | .854 |  | .668131 | .208622 | .014 |  | -.072057 | .065907 | .303 |  | .195653 | .375023 | .602 |  |
| Intercept | 2.952133 | .054529 | .000 |  | .712666 | .145954 | .001 |  | 7.524138 | .044263 | .000 |  | 10.03472 | .276347 | .000 |  |
|  |  |  |  |  |  |  |  |  |  |  |  |  |  |  |  |  |
| Maximal model |  |  |  | 295.532 |  |  |  | 452.488 |  |  |  | 170.330 |  |  |  |  |
| DNA | .165297 | .541761 | .761 |  | -.379425 | .495825 | .452 |  | -.758756 | .575605 | .315 |  | -.805879 | .536391 | .253 | 189.339 |
| CD | -.032034 | .667317 | .962 |  | -.556693 | .613948 | .374 |  | .557156 | .707474 | .511 |  | .703424 | .662568 | .384 |  |
| Organic layer | .051076 | .715182 | .943 |  | .651095 | .658527 | .333 |  | -.971855 | .757957 | .325 |  | -.924150 | .710574 | .305 |  |
| DNA × CD | -.043797 | .725981 | .952 |  | -1.134452 | .666657 | .103 |  | .485964 | .770220 | .591 |  | .205198 | .720087 | .799 |  |
| DNA × OL | .068464 | .816115 | .933 |  | .588265 | .748626 | .440 |  | -.987034 | .866179 | .370 |  | -.840514 | .809110 | .393 |  |
| DNA × CD × OL | -.228211 | 1.08679 | .834 |  | -1.489623 | 1.007391 | .153 |  | .778558 | 1.148808 | .566 |  | .835965 | 1.083674 | .509 |  |
| Intercept | 3.279439 | .525818 | .000 |  | .667538 | .483119 | .181 |  | 6.999621 | .557815 | .006 |  | 2.536334 | .521729 | .028 |  |

^1^response variable was ln transformed
